# Long‐Term Evaluation of Givinostat in Duchenne Muscular Dystrophy, and Natural History Comparisons

**DOI:** 10.1002/acn3.70165

**Published:** 2025-08-19

**Authors:** Craig M. McDonald, Michela Guglieri, Dragana Vučinić, Gyula Acsadi, John F. Brandsema, Claudio Bruno, Erika L. Finanger, Amy Harper, Mercedes Lopez Lobato, Riccardo Masson, Nuria Muelas, Francina Munell, Yoram Nevo, Yann Péréon, Han Phan, Valeria A. Sansone, Mariacristina Scoto, Tracey Willis, Richard S. Finkel, Krista Vandenborne, Sara Cazzaniga, Silvia Montrasio, Federica Alessi, Paolo Bettica, Eugenio Mercuri, the Givinostat Study 51 Investigators, the Cooperative International Neuromuscular Research Group (CINRG) Duchenne Natural History Study (DNHS) Investigators, the ImagingDMD Investigators, Enrico Bertini, Giacomo Pietro Comi, Eugenio Maria Mercuri, Giuseppe Vita, Sonia Messina, Claudio Bruno, Riccardo Masson, Valeria Sansone, Nathalie Goemans, Liesbeth De Waele, Laurent Servais, Teresa Gidaro, Odile Boespflug‐Tanguy, Yann Péréon, Jessika Johannsen, Astrid Blaschek, Ulrike Schara‐Schmidt, Erik Niks, Imelda de Groot, Saskia Houwen‐van Opstal, Andres Nascimento, Juan Jesus Vilchez, Nuria Muelas, Francina Munell, Marcos Madruga Garrido, Mercedes Lopez Lobato, Michaela Guglieri, Tracey Willis, Stefan Spinty, Daniel Hawcutt, Mariacristina Scoto, Jean K. Mah, Laura McAdam, Kathryn Selby, Katherine Mathews, Craig McDonald, Craig Zaidman, Barry Byrne, John Brandsema, Gyula Acsadi, Chamindra Laverty, Amy Harper, Erika Finanger, Han Phan, Yoram Nevo, Vedrana Milic Rasic, Dragana Vucinic

**Affiliations:** ^1^ University of California Davis Health Sacramento California USA; ^2^ The John Walton Muscular Dystrophy Research Centre, Translational and Clinical Research Institute Newcastle University and Newcastle Hospitals NHS Foundation Trust Newcastle upon Tyne UK; ^3^ Clinic for Neurology and Psychiatry for Children and Youth Belgrade Serbia; ^4^ Division of Neurology Connecticut Children's Hartford Connecticut USA; ^5^ Division of Neurology The Children's Hospital of Philadelphia Philadelphia Pennsylvania USA; ^6^ Center of Translational and Experimental Myology IRCCS Istituto Giannina Gaslini Genova Italy; ^7^ Department of Neuroscience, Rehabilitation, Ophthalmology, Genetics, Maternal and Child Health – DINOGMI University of Genova Genova Italy; ^8^ Shriners Hospitals for Children – Portland Portland Oregon USA; ^9^ Division of Neurology Virginia Commonwealth University, Children's Hospital of Richmond Richmond Virginia USA; ^10^ Hospital Universitario Virgen del Rocio Sevilla Spain; ^11^ Developmental Neurology Unit Fondazione IRCCS Istituto Neurologico Carlo Besta Milan Italy; ^12^ Neuromuscular Diseases Unit, Neurology Department Hospital Universitari i Politècnic La Fe Valencia Spain; ^13^ Neuromuscular Reference Centre, European Reference Network for Neuromuscular Diseases (ERN‐EURO‐NMD), Neuromuscular and Ataxias Research Group Instituto de Investigación Sanitaria La Fe Valencia Spain; ^14^ Centro de Investigación Biomédica en Red de Enfermedades Raras (CIBERER), U763 Valencia Spain; ^15^ Pediatric Neuromuscular Diseases Unit, Pediatric Neurology Section, Pediatric Department Hospital Universitari Vall d'Hebron, European Reference Network for Neuromuscular Diseases (ERN‐EURO‐NMD) Barcelona Spain; ^16^ Institute of Neurology – Schneider Children's Medical Center of Israel Tel‐Aviv University Tel‐Aviv Israel; ^17^ CHU Nantes, Reference Centre for Neuromuscular Disorders AOC FILNEMUS, Euro‐NMD Hôtel‐Dieu Nantes France; ^18^ MD Rare Disease Research, LLC Atlanta Georgia USA; ^19^ Neurorehabilitation Unit, The NeMO Clinical Center in Milan University of Milan Milan Italy; ^20^ Dubowitz Neuromuscular Centre and MRC Centre for NMD Great Ormond Street Hospital & UCL Great Ormond Street Institute of Child Health London UK; ^21^ The Robert Jones and Agnes Hunt Orthopaedic Hospital NHS Foundation Trust Oswestry UK; ^22^ Center for Experimental Neurotherapeutics St. Jude Children's Research Hospital Memphis Tennessee USA; ^23^ Division of Neurology Nemours Children's Hospital Orlando Florida USA; ^24^ ImagingDMD and Department of Physical Therapy University of Florida Gainesville Florida USA; ^25^ Italfarmaco SpA Milan Italy; ^26^ Pediatric Neurology Universita Cattolica del Sacro Cuore Rome Italy; ^27^ Centro Clinico Nemo Fondazione Policlinico Gemelli IRCCS Rome Italy

**Keywords:** Duchenne muscular dystrophy, efficacy, long term, safety

## Abstract

**Objectives:**

This ongoing, open‐label extension study is evaluating the long‐term safety, tolerability, and efficacy of givinostat, a Class I and II histone deacetylase inhibitor, in patients with Duchenne muscular dystrophy (DMD).

**Methods:**

The recruited patients completed one of two prior clinical studies (one Phase 2 and one Phase 3 [EPIDYS]), receiving givinostat or placebo, or were successfully screened but not randomized into EPIDYS. All receive givinostat oral suspension open‐label at a flexible, weight‐based dose in addition to systemic corticosteroids, and attend visits every 4 months.

**Results:**

A total of 194 patients are included in the current analyses, with a mean duration of givinostat exposure (excluding use in prior studies) of 559.6 days (SD 373.0); when including use in the prior studies, the maximum exposure to givinostat was > 8 years. Although the majority of patients reported ≥ 1 adverse event (169/194 [87.1%]), most were mild/moderate in severity, and the safety profile of givinostat was consistent with prior studies. Post hoc comparisons with natural history datasets (ImagingDMD and CINRG) suggest, in propensity matched populations, givinostat added to systemic corticosteroids significantly delayed the loss of the ability to rise from the floor, the loss of the ability to complete the 4‐stair climb test, and the loss of ambulation (by medians of 2.0–3.3 years; all nominal *p* < 0.05).

**Interpretation:**

Overall, the safety and tolerability of long‐term administration of givinostat in patients with DMD was consistent with previous studies. Comparisons with natural history data suggest that givinostat delays the occurrence of major disease progression milestones.

**Trial Registration:**

EudraCT number: 2017‐000397‐10; ClinicalTrials.gov identifier: NCT03373968

## 1 Introduction

Duchenne muscular dystrophy (DMD), the most common childhood muscular dystrophy [1], is a lethal, X‐linked neuromuscular disorder caused by complete deficiency of the protein dystrophin due to loss‐of‐function mutations in the *DMD* gene. Dystrophin is part of the dystrophin‐associated protein complex that supports the integrity of the sarcolemma from mechanical stress caused by routine muscle contraction [1, 2], and regulates histone deacetylase (HDAC) activity [3]. Pathologic upregulation of HDAC occurs in DMD and increases inflammation, muscle fiber degeneration, adipogenesis, and fibrosis, and impairs muscle fiber regeneration through epigenetic pathways and altered gene expression [4, 5, 6, 7, 8]. In dystrophin's absence, muscle fibers undergo mechanical injury leading to impaired repair, inflammation, and necrosis, subsequently being replaced by fatty and fibrotic tissue [9, 10]. This results in muscle wasting and weakness, functional decline, loss of ambulation, and death due to cardiac or respiratory failure [9, 10].

Givinostat (DUVYZAT; Italfarmaco SpA, Milan, Italy) is a Class I and II HDAC inhibitor that counteracts the pathogenic events downstream of dystrophin deficiency. It is the first nonsteroidal treatment for DMD irrespective of the specific underlying genetic variant [11] and has been approved in the United States and the United Kingdom for the treatment of DMD in patients aged ≥ 6 years, regardless of genetic mutation [12]. In preclinical studies, givinostat had anti‐inflammatory properties associated with anti‐fibrotic and pro‐regenerative activities [13, 14, 15, 16]. Clinically, givinostat has been investigated in boys with DMD in two clinical studies—a Phase 2 study, in which treatment with givinostat in addition to systemic corticosteroids increased muscle fiber area fraction and reduced tissue fibrosis, necrosis, and fatty replacement in muscle biopsies [17]; and a Phase 3 study (EPIDYS) where givinostat, again in addition to systemic corticosteroids, delayed disease progression compared with placebo, as measured by the 4‐stair climb (4SC), with some evidence of a delay in other clinical endpoints and a reduction in vastus lateralis fat fraction measured by magnetic resonance spectroscopy [18]. Given the progressive nature of DMD, and the expectation that givinostat treatment will be lifelong in DMD, an open‐label extension study is ongoing that aims to evaluate the long‐term safety and tolerability of givinostat, and to continue monitoring signs of efficacy in this population. The patients recruited completed one of the two prior clinical studies (or were successfully screened for entry into EPIDYS, but were not randomized as recruitment closed). Since all patients in the extension study received givinostat open‐label, data were compared to two natural history datasets.

## 2 Methods

**FIGURE 1:**
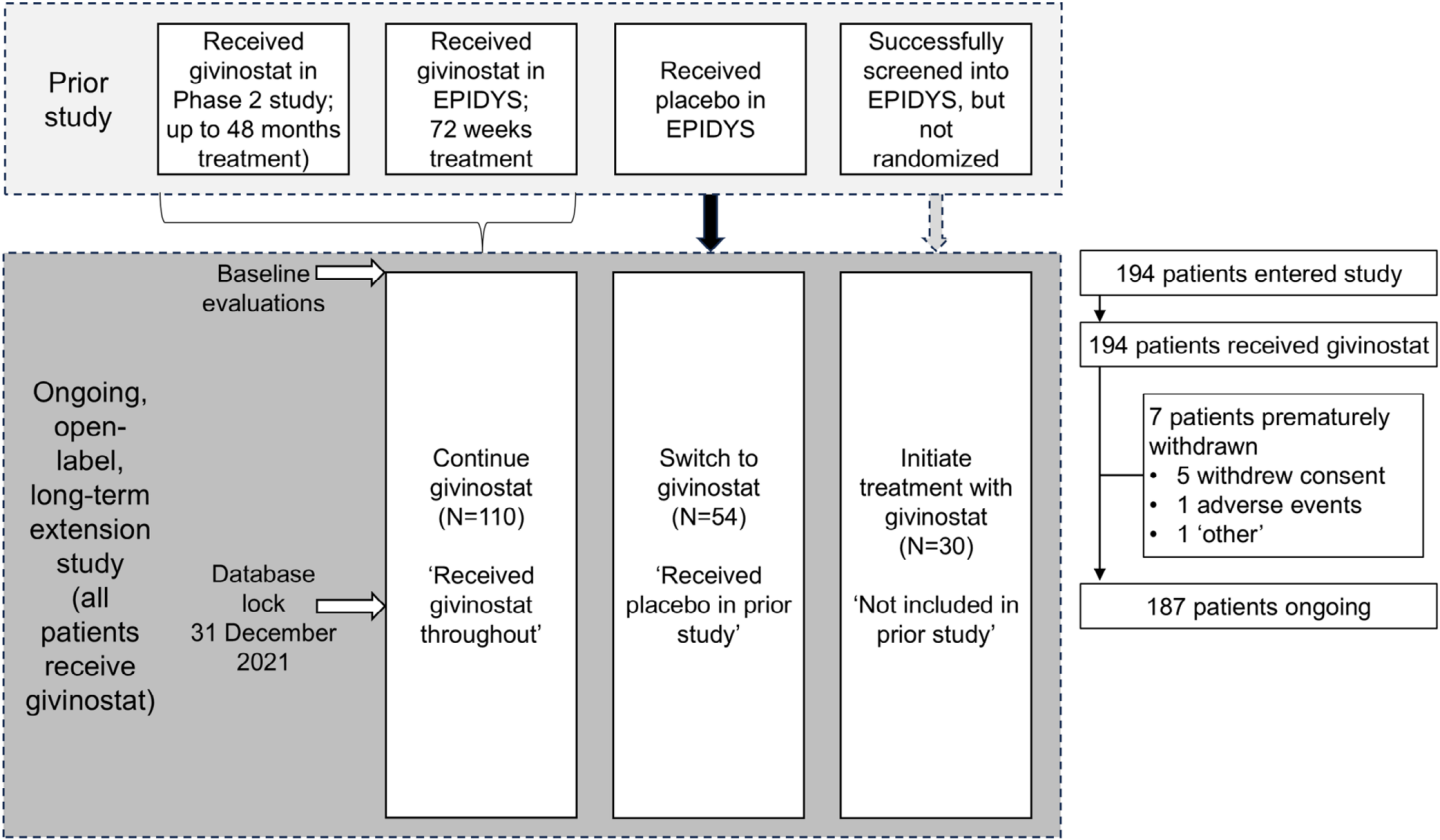
Study design and patient flow through the study.

This international, multicenter, single‐arm, open‐label extension study commenced recruitment on 24 October 2017. This manuscript uses data from a database lock of 31 December 2021. The extension study was open to males aged ≥ 6 years, who (Figure 1):

- Completed one of the prior DMD studies receiving givinostat [17, 18]. On entering the open‐label extension, these patients continued givinostat at the dose being taken on completion of the prior study.
- Completed EPIDYS receiving placebo [18]. On entering the open‐label extension, these patients switched to givinostat at a dose based on the volume of placebo received at the end of EPIDYS (Table S1; e.g., if the patient received 2.0 mL twice daily [bid] of placebo on completion of EPIDYS, they received 2.0 mL bid of givinostat, i.e., 20.0 mg, on entry to the open‐label extension).
- Met the inclusion/exclusion criteria for entry into EPIDYS, but were not randomized as recruitment into Group B had completed due to the participant cap being reached [18]. On entering the open‐label extension, these patients initiated givinostat at the “treatment naïve” dose, which was the lower of the two EPIDYS starting doses (Table S1).

For the follow‐up duration, patients receive givinostat oral suspension open‐label at a flexible, weight‐based dose (Table S1). Details of givinostat dose changes and stopping rules are in the Supporting Information, along with the full inclusion and exclusion criteria. All patients also receive systemic corticosteroids, with dose adjustments following standard care and not influenced by the study protocol.

Patients joining from EPIDYS had platelet count assessments weekly during the first month of the open‐label extension study, then every 2 weeks during the second month. All patients (including those joining from the Phase 2 study) attend visits every 4 months, undergoing physical examinations, vital signs, electrocardiogram (with central reading), and blood and urine sample assessments, with echocardiogram assessed yearly. Ambulant patients complete the following every 4 months: timed‐function tests (timed rise from the floor [TTRF], 4‐stair climb [4SC] [19, 20], and time to walk/run 10 m [10MWT]), North Star Ambulatory Assessment (NSAA) [21, 22, 23], 6‐min walk test (6MWT) [19, 24], and knee extension and elbow flexion strength by hand‐held myometry evaluations. All patients complete the following every 4 months: Performance of the Upper Limb (PUL), Motor Function Measure (MFM). In addition, all patients complete a quality‐of‐life questionnaire annually (Pediatric Quality of Life Inventory [PedsQL]). Pulmonary function tests (forced vital capacity [FVC] and peak expiratory flow [PEF]) are completed annually by ambulant patients and every 4 months by non‐ambulant patients. Finally, non‐ambulant patients complete the Egen Klassifikation [25] and Barthel Index [26] physical function evaluations and elbow flexion strength every 4 months. All functional and strength assessments are evaluated by qualified and trained functional evaluators (i.e., physiotherapists).

All patients and their parent/legal guardian provided written informed consent prior to open‐label extension study entry. The study was approved by the independent ethics committees at each institution, is being performed in accordance with the Declaration of Helsinki and Good Clinical Practice. By the database lock, the protocol had been amended three times (see Supporting Information).

### 2.1 Outcomes

The primary objective is to assess the long‐term safety and tolerability of givinostat in ambulant and non‐ambulant patients with DMD. Secondary efficacy endpoints in ambulant patients are change from baseline at the end of each year in timed‐function tests, NSAA, 6MWT, and muscle strength by hand‐held myometry. Secondary efficacy endpoints in all patients include: change from baseline at the end of each year in PUL, MFM, respiratory function, and PedsQL. Changes from baseline in the following secondary efficacy endpoints are being evaluated in non‐ambulant patients: Egen Klassifikation, Barthel Index, and elbow flexion strength by hand‐held myometry. As exploratory outcomes, the efficacy endpoints at the 24‐month timepoint were compared descriptively between patients who received givinostat in the prior studies and those who previously received placebo to explore the effect of delayed treatment initiation.

### 2.2 Sample Size and Statistical Methods

This open‐label extension study is not formally powered and no sample size was calculated. Multiplicity control was not applicable. Handling of missing data is described in the Supporting Information.

The primary (safety) endpoint (type, incidence, and investigator‐assessed severity of adverse events) is evaluated descriptively, with the secondary efficacy endpoints reported descriptively in terms of changes from baseline. The exploratory efficacy outcomes were analyzed using a repeated‐measures analysis of covariance (ANCOVA) model, with change from baseline in each variable as dependent variable, terms for baseline value and age at first dose in the current open‐label extension study as covariates, and “treatment group” (i.e., “received givinostat throughout,” “received placebo in prior study,” and “received givinostat throughout”) and concomitant corticosteroid use (prednisolone, deflazacort, or other) as independent class variables. The exploratory outcomes were not analyzed in non‐ambulant patients, as data were available for < 10 patients per group.

The safety population, used for all safety analyses, is all patients who received at least one dose of study medication. The efficacy population includes all enrolled patients who received at least one dose of study medication and who provided at least one post‐baseline efficacy measurement; it is used for all efficacy analyses.

Post hoc analyses were conducted to compare patient‐level data from patients who either received givinostat in EPIDYS or who were successfully screened for entry into EPIDYS but enrolled directly in this open‐label extension study (i.e., excluding those who received placebo in EPIDYS or who took part in the Phase 2 study) with those of two natural history disease studies, the Cooperative International Neuromuscular Research Group (CINRG) Duchenne Natural History Study (DNHS) [27, 28], and the ImagingDMD cohort [29]. Patients from the natural history datasets were included in the analyses if they met the key inclusion criteria of EPIDYS—in particular, ambulant males aged ≥ 6 years with a DMD diagnosis, baseline 4SC ≤ 8 s, TTRF ≥ 3 and < 10 s, and receiving stable corticosteroid treatment. Patients from the natural history datasets were excluded if they were receiving any investigational drug or any dystrophin restoration product (such as ataluren).

A propensity score analysis [30, 31, 32, 33] was implemented to establish a control group composed of matched natural history data comparable to the givinostat group. Patients' functional status at a given timepoint together with corticosteroid treatment are the best predictors of the trajectory of the change over time in function tests [34, 35, 36]. Therefore, the following baseline covariates were considered in the matching approach: 4SC, TTRF, 10MWT, and corticosteroid use (deflazacort, other). Nearest available matching (nearest neighbor), based on the closest propensity score (on logit scale), was performed using a greedy approach to match each trial patient to one natural history patient. A caliper of 0.5 was used (chosen high to ensure the majority of patients in the givinostat arm had a match in the natural history data). The following disease milestone endpoints were included in these analyses: persistent (i.e., at ≥ 2 consecutive visits) loss of rise from floor, loss of ability to perform 4SC, and loss of ambulation (see the Supporting Information for definitions), based on published clinically meaningful milestones previously used to assess the long‐term impact of corticosteroids [27]. Kaplan–Meier estimates of the median age at these disease milestones were calculated for each group, with the associated two‐sided 95% confidence intervals (CI). Hazard ratios and associated two‐sided 95% CIs for the givinostat versus control comparisons were determined using Cox proportional hazards modeling, including randomized treatment group as independent classification factor. Two sets of sensitivity analyses were run: the first included all patients; the second included only patients treated with deflazacort. For these post hoc analyses, the baseline values for patients receiving givinostat were taken prior to givinostat initiation.

## 3 Results

### 3.1 Participants

A total of 194 patients are included in the current analyses, the first entering this open‐label extension on 24 October 2017. Seven had withdrawn by the database lock (31 December 2021; five withdrew consent, one due to an adverse event, and one with the reason recorded as “other;” Figure 1). A total of 146 patients entered after completing EPIDYS (92 receiving givinostat throughout [“received givinostat throughout”] and 54 switching from placebo [“received placebo in prior study”]), 18 entered from the Phase II study (all receiving givinostat throughout), and 30 met the EPIDYS inclusion/exclusion criteria but were not randomized as recruitment had stopped and so entered the extension directly (“not included in prior study”). Mean ages on entry to the open‐label extension study were 11.6, 11.4, and 10.5 years in the received givinostat throughout, received placebo in prior study, and not included in prior study groups, respectively, with most white, and the most common DMD mutations being large deletions (Table 1).

**TABLE 1:** Baseline demographics and disease characteristics (safety population).

Parameter	Received givinostat throughout^a^ (*N* = 110)	Received placebo in prior study^a^ (*N* = 54)	Not included in prior study^a^ (*N* = 30)
Age, years, mean (SD)	11.6 (2.09)	11.4 (2.04)	10.5 (2.25)
Race, *n* (%)			
White	100 (90.9)	50 (92.6)	22 (73.3)
Asian	2 (1.8)	2 (3.7)	2 (6.7)
Black	4 (3.6)	0	1 (3.3)
Other	4 (3.6)	2 (3.7)	5 (16.7)
BMI, kg/m^2^, mean (SD)	20.8 (4.69)	21.1 (5.12)	20.2 (4.05)
Ambulant at screening, *n* (%)	102 (92.7)	51 (94.4)	30 (100)
Time since diagnosis, years, mean (SD)	7.5 (2.57)	7.2 (2.78)	6.9 (2.74)
DMD mutation, *n* (%)			
Deletion	71 (64.5)	36 (66.7)	17 (56.7)
Duplication	13 (11.8)	8 (14.8)	6 (20.0)
Point mutation	17 (15.5)	2 (3.7)	4 (13.3)
Other	8 (7.3)	8 (14.8)	3 (10.0)
Concomitant corticosteroid regimen, *n* (%)^b^			
Prednisone daily	7 (6.4)	8 (14.8)	4 (13.3)
Prednisone intermittent	9 (8.2)	4 (7.4)	0
Deflazacort daily	82 (74.5)	37 (68.5)	26 (86.7)
Deflazacort intermittent	12 (10.9)	7 (13.0)	1 (3.3)

Abbreviations: BMI, body mass index; DMD, Duchenne muscular dystrophy.

^a^ All patients were also receiving systemic corticosteroids for the full duration of the follow‐up period.

^b^ Note that although all patients took systemic corticosteroids for the full follow‐up duration, the regimen could change, and so three patients are counted twice.

At database lock, the mean givinostat exposure duration in this open‐label extension study (excluding use in the prior studies) was 615.6 (SD 446.0 [maximum 1529]), 505.8 (SD 272.1 [maximum 940]), and 450.9 (SD 116.0 [maximum 608]) days in the givinostat throughout, prior placebo, and not included groups, respectively, with an overall mean exposure of 559.6 days (over 18 months). Including use in the prior studies, the overall maximum exposure to givinostat in the givinostat throughout group is in excess of 8 years. The givinostat dose was modified at least once during the open‐label extension study in 68/110 (61.8%), 37/54 (68.5%), and 14/30 (46.7%) of patients in the givinostat throughout, prior placebo, and not included groups, respectively, with 50/110 (45.5%), 15/54 (27.8%), and 8/30 (26.7%) increasing the dose (all due to weight gain), and 20/110 (18.2%), 25/54 (46.3%), and 5/30 (16.7%) decreasing the dose (mainly due to adverse events). Givinostat treatment was permanently stopped by 4/110 patients (3.6%) in the givinostat throughout group (one due to an adverse event; three withdrew consent), and 2/54 (3.7%) in the prior placebo group (both due to adverse events).

### 3.2 Safety

The majority of patients reported at least one adverse event (169/194 [87.1%]); most were mild or moderate in severity (according to the National Cancer Institute Common Terminology Criteria for Adverse Events, version 4.03), and few were serious, with overall, severe and serious adverse events equally distributed among treatment groups (Table 2). The most common adverse events in the givinostat throughout group were increases in blood triglyceride levels, falls, and diarrhea. A smaller proportion of patients experienced diarrhea in this group than in either of the other groups, suggesting this event becomes less common on prolonged use (given these patients have received givinostat for longer than the other two groups, when considering use in the prior studies). There is no clear explanation for the incidence of falls (except that patients receiving givinostat perhaps retained mobility for longer), but none were classified as severe or serious. The most common adverse events related to givinostat were blood triglyceride increases, platelet count decreases, and diarrhea. Adverse events leading to dose reduction were experienced by 16/110 (14.5%), 15/54 (27.8%), and 4/30 (13.3%) of patients in the givinostat throughout, prior placebo, and not included groups, respectively.

**TABLE 2:** Adverse events, overall and most common preferred terms (≥ 10% in the overall population for adverse events; ≥ 5% for treatment‐related adverse events; ≥ 2 patients for adverse events leading to treatment discontinuation, study withdrawal, and serious or severe adverse events).

Patients (%)	Received givinostat throughout^a^ (*N* = 110)	Received placebo in prior study^a^ (*N* = 54)	Not included in prior study^a^ (*N* = 30)
Adverse event	96 (87.3)	47 (87.0)	26 (86.7)
Blood triglycerides increased or hypertriglyceridemia	23 (20.9)	11 (20.4)	2 (6.7)
Fall	22 (20.0)	5 (9.3)	4 (13.3)
Diarrhea	20 (18.2)	15 (27.8)	11 (36.7)
Platelet count decreased or thrombocytopenia	19 (17.3)	19 (35.2)	5 (16.7)
Pyrexia	19 (17.3)	7 (13.0)	2 (6.7)
Headache	16 (14.5)	9 (16.7)	6 (20.0)
Vomiting	16 (14.5)	8 (14.8)	5 (16.7)
Abdominal pain	12 (10.9)	9 (16.7)	1 (3.3)
Adverse event considered related to treatment	60 (54.5)	36 (66.7)	18 (60.0)
Blood triglycerides increased or hypertriglyceridemia	21 (19.1)	10 (18.5)	2 (6.7)
Platelet count decreased or thrombocytopenia	19 (17.3)	18 (33.3)	5 (16.7)
Diarrhea	10 (9.1)	9 (16.7)	10 (33.3)
Abdominal pain	5 (4.5)	5 (9.3)	1 (3.3)
Severe adverse event	13 (11.8)	5 (9.3)	3 (10.0)
Femur fracture	3 (2.7)	1 (1.9)	0
Back pain	2 (1.8)	1 (1.9)	0
Blood triglycerides increased or hypertriglyceridemia	1 (0.9)	1 (1.9)	0
Serious adverse event	12 (10.9)	8 (14.8)	3 (10.0)
Femur fracture	3 (2.7)	3 (5.6)	0
Tendinous contracture	2 (1.8)	0	0
Back pain	1 (0.9)	1 (1.9)	0
Dehydration	1 (0.9)	1 (1.9)	0
Serious adverse event considered related to treatment	0	1 (1.9)	0
Life‐threatening or fatal adverse event	0	0	0
Adverse event leading to study treatment discontinuation	1 (0.9)	2 (3.7)	0
Adverse event leading to study withdrawal	1 (0.9)	2 (3.7)	0

Abbreviation: COVID‐19, coronavirus disease 2019.

^a^ All patients were also receiving systemic corticosteroids for the full duration of the follow‐up period. The severity of the adverse events was assessed and graded by the investigators according to the National Cancer Institute Common Terminology Criteria for Adverse Events (version 4.03).

At database lock, there had been no fatal or life‐threatening events. All three patients with adverse events resulting in treatment discontinuation subsequently withdrew from the study. The patient who received givinostat throughout withdrew due to nausea, considered by the investigator to be moderate in severity. The other two previously received placebo, with one withdrawing due to an adverse event reported as increased blood triglycerides (mild in severity), and the other due to atrial fibrillation (the only treatment‐related severe adverse event). All three events resolved (the atrial fibrillation was new‐onset, in a patient with a history of hypertrophic cardiomyopathy, and resolved with treatment prior to givinostat discontinuation).

**FIGURE 2:**
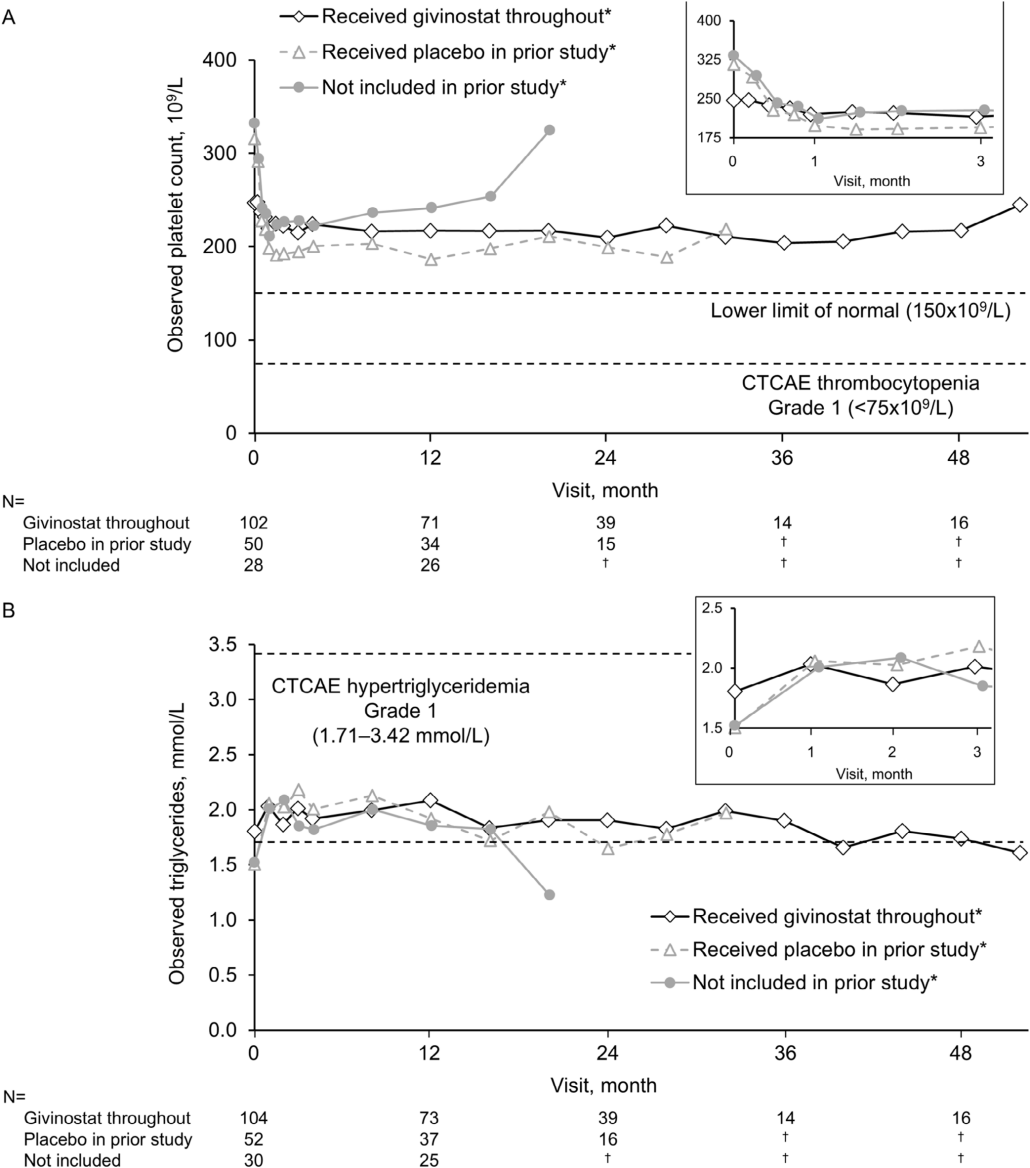
Mean observed values for (A) platelet counts and (B) triglyceride concentrations. *All patients were also receiving systemic corticosteroids for the full duration of the follow‐up period. ^†^No patients have reached this timepoint. *N* indicates the number available for assessment. CTCAE, Common Terminology Criteria for Adverse Events.

For platelet counts, in the two groups that initiated givinostat on entry to the open‐label extension study (i.e., prior placebo and not included) there was a decrease from baseline in the initial 2 weeks, with the value then remaining stable (Figure 2A). In the group of patients who received givinostat in the prior studies, the baseline value was lower than the other two groups, with mean values remaining largely unchanged over follow‐up. Five of the 110 patients (4.5%) who received givinostat throughout reduced givinostat dose due to low platelet counts, compared to 4/54 (7.4%) and 1/30 (3.3%) in the prior placebo and not included groups, respectively; none of whom had any overt clinical manifestations (Table S2). Most other hematology values were generally within the normal range throughout the open‐label extension study follow‐up period.

There was an initial increase from baseline in mean triglyceride concentration in the two groups initiating givinostat on entry to the open‐label extension study (Figure 2B). Two (of 109 [1.8%]) and 1/53 (1.9%) patients in the givinostat throughout and prior placebo groups, respectively, decreased givinostat dose due to increased triglyceride levels (Table S2). Mean creatinine values remained similar to the open‐label extension study baseline value for the duration of follow‐up in the givinostat throughout group (Figure S1). In the prior placebo and not included groups, values increased from baseline at the Week 4 visit by approximately 20% and 10%, respectively, then remained stable over follow‐up. Most of the other abnormal blood chemistry values were consistent with those commonly observed in patients with DMD. There were no clinically meaningful changes in urinalysis values or in vital signs assessments, with mean cystatin C values unchanged (Figure S2).

No patients had an increase from baseline > 60 ms in QTc Fridericia's correction (QTcF) interval or a QTcF interval > 450 ms. There were also no clinically relevant echocardiography findings at baseline or during follow‐up. For example, baseline left ventricular ejection fraction (LVEF) and left ventricular end diastolic diameter (LVEDD) dimension values were similar in the three groups (means of 60.4%–63.6% for LVEF and 40.0–40.6 mm for LVEDD) and remained largely unchanged over follow‐up (Table S2).

### 3.3 Efficacy

#### 3.3.1 Ambulant Patients

Most recruited patients were still ambulant on entry to the open‐label extension study (183/194 [94.3%]). Overall, there were declines from baseline over follow‐up in all three groups of patients for TTRF (velocity and time), 4SC (velocity and time), 10MWT (velocity and time), NSAA total score, 6MWT, and muscle strength (Figures S3–S14).

In the exploratory efficacy comparisons of the givinostat throughout versus prior placebo groups, there were no between‐group differences in any of the endpoints at 24 months, suggesting that DMD progression was similar in the two groups (Table S3). For example, the givinostat throughout versus prior placebo least squares means differences at 24 months were 0.02 tasks/s (95% confidence interval −0.177, 0.222) for 4SC velocity, and −1.65 (−5.181, 1.882) for NSAA.

#### 3.3.2 All Patients

There were declines from baseline over follow‐up in PUL (total and domain scores) and MFM (total and dimension scores) in the givinostat throughout and prior placebo groups (Figures S15–S22). In the “not included” group (who were on average younger on entry to the open‐label extension study than the other two groups), baseline values indicated more preserved baseline function, with less decline following initiation of givinostat than the other groups—although this group included the fewest patients, with follow‐up only available to Month 12. There was a decline in FVC percent predicted over the follow‐up period in all groups, with PEF percent predicted remaining largely unchanged (Figures S23 and S24).

Quality of life evaluated with PedsQL total score was consistent with the functional assessments, with a gradual decline in mean scores in the subgroups of patients who had previously received givinostat and placebo, whereas values were stable in the not included group (Figure S25). As with the analyses in the ambulant patients, in the exploratory comparisons of the givinostat throughout versus prior placebo groups, there were no differences in any of the endpoints at 24 months (Table S3).

#### 3.3.3 Non‐Ambulant Patients

Eleven patients were non‐ambulant on entry to the open‐label extension study (all completed EPIDYS); eight received givinostat throughout, and three previously received placebo. This small number resulted in high variability in the assessed parameters, with limited changes from baseline (Figures S26–S29).

### 3.4 Comparison of Disease Progression Milestones With Natural History Datasets

A total of 148 patients receiving givinostat were included in the comparisons with the natural history datasets, 118 of whom received givinostat in EPIDYS, with 30 patients entering the open‐label extension directly. A total of 197 comparator patients from the natural history datasets were included in the analyses (Table S4).

After propensity matching (using the criteria listed in Section 2.2), 142 patients receiving givinostat and 142 from the natural history cohort were included in the analyses (Table 3). The propensity score matching was considered effective, with a logit score standardized mean difference of 0.23 (< 0.1) and a variance ratio of 1.66 (close to 1), with similar results for the covariates. Importantly, after propensity matching, results from the four function tests at baseline were well matched between the givinostat and control groups, although patients in the givinostat group were older and had a longer time since diagnosis (Table 3).

**TABLE 3:** Demographic and baseline characteristics, matching patients from the givinostat study population and the natural history datasets (control group).

	Givinostat^a^ (*N* = 142)	Natural history cohort^a^ (*N* = 142)
Age, years, mean (SD)	9.9 (2.09)	8.1 (1.76)
Race, *n* (%)		
Asian	6 (4.2%)	9 (6.3%)
Black	4 (2.8%)	1 (0.7%)
White	122 (85.9%)	73 (51.4%)
Other/unknown	10 (7.0%)	7 (4.9%)
Missing^b^	0	52 (36.6%)
Body mass index, kg/m^2^, mean (SD)	19.7 (4.04)	18.4 (3.86)
Time since diagnosis^b^, years, mean (SD)	5.8 (2.63)	4.4 (1.77) (*N* = 52)
Use of corticosteroids, *n* (%)		
Deflazacort	114 (80.3%)	92 (64.8%)
Other	28 (19.7%)	50 (35.2%)
Time to rise from floor, s, mean (SD)	5.8 (2.12)	5.3 (1.76)
4‐stair climb, s, mean (SD)	3.5 (1.22)	3.7 (1.15)
Time to walk/run 10 m, s, mean (SD)	5.5 (1.28)	5.6 (1.69)
6‐min walk distance,^b^ m, mean (SD)	400 (68.4)	383 (56.8) (*N* = 49)

^a^ All patients were also receiving systemic corticosteroids for the full duration of the follow‐up period.

^b^ Information on race is not available from ImagingDMD, with information on time since diagnosis and 6‐min walk distance not available from CINRG.

**FIGURE 3:**
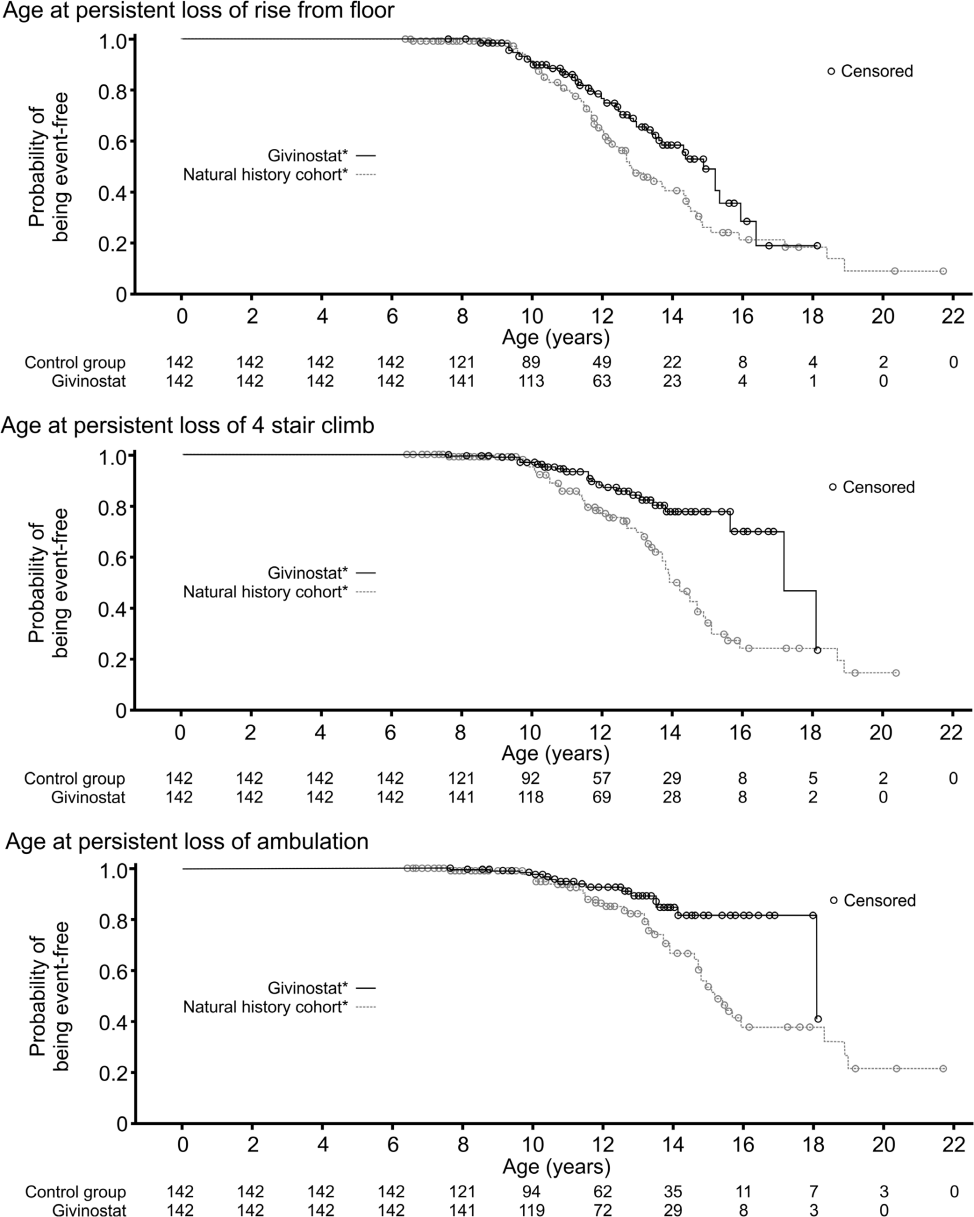
Outcome analyses, matching patients from the givinostat study population and the natural history datasets. *All patients were also receiving systemic corticosteroids for the full duration of the follow‐up period.

Overall, patients receiving givinostat had a delay in disease progression compared to the natural history cohort in terms of all three assessed endpoints (Table 4 and Figure 3). These data suggest that the addition of givinostat to standard of care systemic corticosteroids can delay the loss of the ability to rise from the floor by a median 2.0 years (hazard ratio [HR] givinostat vs. natural history 0.66 [95% confidence interval 0.45, 0.96]; nominal *p* = 0.028), the ability of 4SC by 3.3 years (HR 0.39 [0.24, 0.65]; nominal *p* < 0.001), and the ability of ambulation by 2.9 years (HR 0.42 [0.23, 0.76]; nominal *p* = 0.004). Results were similar when the overall population from the givinostat study was compared with all patients in the two natural history datasets (Table S5).

**TABLE 4:** Comparison of the occurrence of major milestones, matching patients from the givinostat study population and the natural history datasets (control group).

	Givinostat^a^ (*N* = 142)	Natural history cohort^a^ (*N* = 142)
Persistent loss of rise from floor, *n* (%)	45 (31.7%)	61 (43.0%)
Age, years, median (95% CI)	14.9 (13.6, 16.0)	12.9 (12.2, 14.3)
Hazard ratio givinostat vs. control (95% CI)	0.66 (0.45, 0.96); *p* = 0.028	
Persistent loss of 4‐stair climb, *n* (%)	21 (14.8%)	52 (36.6%)
Age, years, median (95% CI)	17.2 (15.7, NE)	13.9 (13.5, 14.9)
Hazard ratio givinostat vs. control (95% CI)	0.39 (0.24, 0.65); *p* < 0.001	
Persistent loss of ambulation, *n* (%)	14 (9.9%)	39 (27.5%)
Age, years, median (95% CI)	18.1 (18.1, NE)	15.2 (14.7, 18.3)
Hazard ratio givinostat vs. control (95% CI)	0.42 (0.23, 0.76); *p* = 0.004	

*Note:* n values are the number of patients reaching the disease milestone. The *p* values in this table are nominal.

Abbreviation: NE, not estimable.

^a^ All patients were also receiving systemic corticosteroids for the full duration of the follow‐up period.

Patients in the natural history cohort were less likely to be receiving deflazacort than those receiving givinostat; this difference improved after propensity matching, but there was still a difference between the two populations, and so a further analysis was conducted that included only those in the propensity matched set receiving deflazacort (*N* = 114 in the givinostat group and 92 in the control group; Table S6). Results were again similar to the propensity matched population in this subgroup of patients (Table S7).

## 4 Discussion

The main purpose of this ongoing open‐label extension study is to evaluate the long‐term safety and tolerability of givinostat. Three distinct groups of patients were recruited. The first received givinostat in a prior study, for whom the additional exposure to givinostat was up to 48 months in the Phase II study and extensions [17], and 72 weeks in EPIDYS [18], such that the overall maximum duration of exposure to givinostat is over 8 years. Analyses of this cohort provide information on the safety profile of givinostat on continued administration. The second group received placebo (double‐blind) in EPIDYS, and then initiated givinostat on entry to the current study at an older age (mean 11.4 years) than those who initiated givinostat at the start of EPIDYS (mean 9.8 years [18]). The data for these patients suggest that the safety profile of givinostat is similar regardless of age at initiation. Finally, the third group met the screening criteria for entry to EPIDYS, but as recruitment had been closed they entered the current extension study instead—it is important that the safety profile of givinostat in these patients was consistent with that of patients who received givinostat in either the Phase 2 study or EPIDYS [17, 18].

Most patients in all three groups reported at least one adverse event, although only 23 had serious adverse events, none life‐threatening or fatal at the time of the database lock. The most common adverse events considered related to treatment by the investigator were increases in blood triglyceride levels, decreases in platelet counts, and diarrhea. These are recognized adverse events of givinostat and can generally be managed by dose reduction or interruption. Changes from baseline in platelet counts in patients who were already receiving givinostat on entry to the open‐label extension study were small; in the other two groups, who initiated givinostat on entry to the extension study, mean counts initially decreased but then stabilized after 2–4 weeks of treatment. Importantly, there were no overt clinical manifestations of thrombocytopenia such as excessive bleeding, and thrombocytopenia was uncommon after 8 weeks on therapy. Similarly, mean triglyceride concentrations increased following givinostat initiation in these two groups, stabilizing after 4 weeks, whereas they were relatively stable in the givinostat throughout group. Furthermore, the most frequently reported serious or severe adverse events included femur fracture and back pain, which are common in patients with DMD, especially those receiving systemic corticosteroids [37, 38], with no meaningful changes in the other safety parameters. Overall, therefore, these data build on prior studies suggesting that givinostat has a predictable safety profile and that the flexible dosing regimen (increasing the dose as patients gain weight [e.g., as they grow], and decreasing or interrupting the dose to manage safety parameters) is appropriate in DMD.

A secondary purpose of the extension study is to evaluate the clinical impact of long‐term treatment with givinostat on DMD disease progression assessed by the sequential loss of clinically meaningful milestones. The progressive nature of DMD makes the efficacy data difficult to interpret in isolation, especially given rates of decline in these endpoints vary widely depending on, for example, patients' age and disease state [36, 39, 40]. As an example, ambulant patients' mean NSAA decline from baseline in the current study of 6.12–7.77 units at 24 months (approximately 3–4 units/year) is broadly consistent with the −3.8 to −4.7 units/year in the overall sample from a UK database, but is much lower than the −5.4 to −8.6 units/year in a subgroup at risk of loss of ambulation [39]. Other endpoints such as 6MWT are influenced by age: given DMD is typically diagnosed during early childhood when motor function continues to improve, patients tend to have an initial improvement from baseline, with values then starting to decline [40]. Since the patients in the current analyses had a mean age of 10 years at baseline, the approximately 110 m decline we observed in this endpoint at 24 months (approximately 55 m/year) is broadly consistent with an approximately 60 m decline over 48 weeks for patients aged ≥ 7 years in a previous study [40]. The muscle strength endpoints in the current study are also consistent with those in the previous 48‐week study, in that the decline was more marked in knee extension than in elbow flexion [40]. It is reassuring, however, that the trends were similar in the groups who received givinostat and placebo in the prior studies, with no difference between these two groups in the exploratory comparisons of the efficacy endpoints at 24 months. Although, as with any DMD therapy, it is likely that earlier initiation is better, these data suggest that even initiating givinostat later in the disease course provides benefit to these patients.

As the efficacy of givinostat was previously assessed over 72 weeks in the Phase 3, placebo‐controlled, EPIDYS study [18], it is important to note that the longer term open‐label efficacy evaluation included post hoc comparisons with natural history data as placebo‐controlled trials become ethically difficult after 18 months, and the evaluation of disease progression milestones, such as loss of ambulation, often requires follow‐up beyond 2 years. These comparator data were obtained prospectively in multicenter natural history studies with rigorous protocols and data collection by trained functional evaluators (CINRG and ImagingDMD) [27, 28, 29], with all patients from the natural history datasets selected to meet the key inclusion criteria of EPIDYS, and then subsequently matched with the givinostat population using a propensity score analysis to address the prognostic importance of baseline disease severity assessed by timed‐function tests. These post hoc comparisons suggested that the addition of givinostat to standard of care systemic corticosteroids delays the occurrence of the major clinically meaningful disease progression milestones, with the loss of the ability to rise from the floor delayed from a median age of 12.9 years in the natural history cohort to 14.9 years with givinostat, loss of the ability to complete the 4SC from 13.9 to 17.2 years, and loss of ambulation from 15.2 to 18.1 years. The delay of ambulation with givinostat is favorable even in comparison to a systematic review of 45 studies of patients with DMD, in which the median age at loss of ambulation in patients receiving systemic corticosteroids varied between 11.0 and 13.4 years [41].

A limitation of these data is that results are restricted to the population who met the inclusion/exclusion criteria and voluntarily agreed to continue in the long‐term extension study. In addition, although propensity score matching is a useful way of controlling for bias and achieving pseudo‐randomization, the process still reflects the limitations of an indirect comparison—for example, the two external control natural history databases were not contemporary with the current study. Further, the number of observations for patients beyond the age of 15 years is limited (although as the study is ongoing, it may be possible to generate additional data for older patients). In addition, there was no way to control for corticosteroid regimen (daily vs. intermittent) or for changing dose, regimens, or type of corticosteroid in the long‐term comparison with the natural history datasets—although thus far, the only adjustments to dose have been increases due to weight gain. Finally, not all endpoints in the long‐term extension study were also assessed in the two natural history datasets. This limited the endpoints that could be included in the comparisons—although we believe that those compared reflect three of the main milestones in DMD progression: initially loss of ability to rise from the floor, progressing to loss of ability to complete the 4‐stair climb test, and finally to loss of ambulation. Further, these endpoints are objectively assessed.

Overall, the safety and tolerability profile of long‐term administration of givinostat in patients with DMD was consistent with the results of previous, shorter duration studies. In addition, the efficacy benefit in terms of functional evaluations seen in these prior studies is consistent with the long‐term data from this study, including the natural history comparisons. Patients continue ongoing follow‐up in this open‐label extension study.

## Author Contributions

Study conception/design: C.M.M., S.C., P.B., E.M. Data acquisition: C.M.M., M.G., D.V., G.A., J.F.B., C.B., E.L.F., A.H., M.L.L., R.M., N.M., F.M., Y.N., Y.P., H.P., V.A.S., M.S., T.W., E.M. Analysis supervision: F.A. Study oversight: P.B., S.C., S.M. All authors were involved in data interpretation and the decision to publish, revised the manuscript for important intellectual content, approved the final version, and agree to be accountable for all aspects of the work.

## Conflicts of Interest

In addition to the medical writing support disclosed in the acknowledgments section, the authors have the following conflicts of interest to disclose. Craig M. McDonald has served as a consultant for clinical trials with Astellas Pharma, Avidity Biosciences, Capricor Therapeutics, Catabasis Pharmaceuticals, Edgewise Therapeutics, Entrada Therapeutics, Epirium Bio (formerly Cardero Therapeutics), FibroGen, Italfarmaco, Pfizer, PTC Therapeutics, Hoffman La Roche, Santhera Pharmaceuticals, Sarepta Therapeutics, and Solid Biosciences. He has received research support for clinical trials from Capricor Therapeutics, Catabasis Pharmaceuticals, Edgewise Therapeutics, Epirium Bio, Italfarmaco, Pfizer, PTC Therapeutics, Santhera Pharmaceuticals, Sarepta Therapeutics, and Solid Biosciences. He serves on external advisory boards related to DMD for Capricor Therapeutics, Edgewise Therapeutics, Eli Lilly, Entrada Therapeutics, Italfarmaco, NS Pharma, Percheron, PTC Therapeutics, Sarepta Therapeutics, Santhera Pharmaceuticals, and Solid Biosciences. All are outside the scope of the current manuscript. Michela Guglieri has served on external advisory boards or safety monitoring boards related to DMD for NS Pharma, Roche, Italfarmaco, Santhera Pharmaceuticals, Dyne, and Antisense Therapeutics. She is the principal investigator for clinical trials in DMD sponsored by Italfarmaco, Pfizer, ReveraGen, Roche, and PTC Therapeutics. She has received personal speaker honoraria from Italfarmaco, Dyne, Roche, Novartis, and support for attending meetings and travel (conference registration fee, travel and accommodation) from Novartis and Italfarmaco. She declares grants or contracts with Edgewise and Sarepta, and unpaid leadership roles with the TREAT‐NMD Global Data Systems Oversight Committee (Chair), the Cooperative International Neuromuscular Research Group (CINRG; member of the executive committee), and the DMD Hub UK (principal investigator). All are outside the scope of the current manuscript. Dragana Vučinić has no other conflicts of interest to disclose. Gyula Acsadi serves as the Chair of the Sarepta Safety Monitoring Board. John F. Brandsema declares grants or contracts from Alexion, Astellas, AveXis/Novartis, Biogen, Catabasis, CSL Behring, Cytokinetics, Fibrogen, Genentech/Roche, Janssen, Pfizer, PTC Therapeutics, Sarepta, and Scholar Rock; consulting fees from Alexion, Argenx, AveXis/Novartis, Biogen, Cytokinetics, Dyne, Edgewise, Fibrogen, Genentech/Roche, Janssen, Momenta, NS Pharma, PTC Therapeutics, Sarepta, Scholar Rock, and Takeda; support for attending meetings and/or travel from CureSMA and the Muscular Dystrophy Association; participation on a data safety monitoring board or advisory board for Cure Rare Disease; and that he is a member of the Cure SMA Medical Advisory Council. All are outside the scope of the current manuscript. Claudio Bruno has received honoraria for advisory boards from Roche, Biogen, Novartis, and for conferences from Roche. All are outside the scope of this manuscript. Erika L. Finanger declares grants or contracts from Novartis, Biogen, Scholar Rock, Fibrogen, PTC Therapeutics, Sarepta, Dyne, and NSPharma; consulting fees from Reata, Pfizer, IFT Therapeutics, Novartis, and Sarepta; support for attending meetings and/or travel from the Muscular Dystrophy Association; and participation on a data safety monitoring board or advisory board for Italfarmaco and Edgewise Therapeutics. All are outside the scope of the current manuscript. Amy Harper declares receipt by Virginia Commonwealth University of contracted funding as principal investigator in clinical research studies related to DMD, SMA, FSHD, and LGMD from Novartis, MLBio, Regenxbio, Santhera, Dyne, and Fulcrum, and as coinvestigator for MDStarNet, and compensation for attendance at an advisory board for Sarepta Gene Therapy. In addition, she declares unpaid roles as an executive committee member for CINRG, director of the Children's Hospital of Richmond MDA Clinic, director of the Children's Hospital of Richmond PPMD DMD Certified Center, and a member of the Nominating Committee of the Child Neurology Society. All are outside the scope of the current manuscript. Mercedes Lopez Lobato has no other conflicts of interest to disclose. Riccardo Masson declares receipt of consulting fees from Roche, Biogen, Novartis, and PTC Therapeutics, payment or honoraria for lectures, presentations, speakers bureaus, manuscript writing, or educational events from Roche, Biogen, and Novartis, support for attending meetings and/or travel from Roche and Novartis, and medical writing support from Novartis. All are outside the scope of the current manuscript. Nuria Muelas declares receipt of grant PI21/01532, supported by the Instituto de Salud Carlos III (ISCIII) and co‐funded by the European Union, and grants PROMETEO/2019/075 and PROMETEO/2023/053 from the Valencian Council for Innovation, Universities Science and Digital Society, consulting fees from PTC Therapeutics, Pfizer, Sanofi, Amicus, and Astellas, honoraria for lectures, presentations, speakers bureaus, manuscript writing, or educational events from PTC Therapeutics, Pfizer, Sanofi, and Amicus, support for attending meetings and/or travel from Sanofi, and participation on a data safety monitoring board or advisory board for PTC Therapeutics, Pfizer, Sanofi, Amicus, and Astellas. All are outside the scope of the current manuscript. Francina Munell declares that she was the principal investigator of the Vall d'Hebron University Hospital, Barcelona, site of the current study. She has no other relevant conflicts of interest to disclose. Yoram Nevo declares that he was the principal investigator of Schneider Children's Medical Center, Israel, site of the current study. Outside the scope of the current manuscript, he received a research grant from Pfizer; was principal investigator for clinical trials conducted by Pfizer, PTC, Sarepta, Biomarin, Santera, and multiple CINRG clinical trials; received honoraria for presentations from PTC, Pfizer, Roche, Sarepta, and Neopharm Israel; attended advisory boards for PTC, Sarepta, and Roche; and was an ad hoc advisor for TEVA. In addition, he declares previous unpaid roles as an executive committee member for CINRG and the head of the Israeli pediatric neurology association. Yann Péréon declares honoraria from Italfarmaco SpA for presentation in symposium and for participation in an advisory board. All are outside the scope of the current manuscript. Han Phan declares that travel and lodging were covered by Italfarmaco SpA to present at World Muscle Society, outside the scope of the current manuscript. Valeria A. Sansone declares consulting fees for scientific activities from Roche, Biogen, Novartis, Dyne, Sanofi, and Solid Biosciences; payment for teaching services from Roche, Biogen, Novartis, Dyne, Sanofi, and Solid Biosciences; and participation in advisory boards for Roche, Biogen, Novartis, Dyne, Sanofi, and Solid Biosciences. All are outside the scope of the current manuscript. Mariacristina Scoto has no other conflicts of interest to disclose. Tracey Willis declares consulting fees from PTC Therapeutics, Pfizer, Santhera, and Sarepta, Novartis, Biogen, and Roche; honoraria for symposia from PTC Therapeutics, Biogen, and Novartis; and support for attending meetings and/or travel from Novartis. All are outside the scope of the current manuscript. Richard S. Finkel declares personal compensation for advisory board/data safety monitoring board participation from Astellas, Biogen, Dyne, Genentech, Ionis, Italfarmaco, ReveraGen, Roche, Sarepta, and Scholar Rock; research funding from Biogen, Dyne, Genentech, Genethon, Italfarmaco, Roche, and Sarepta, Scholar Rock; editorial fees from Elsevier for coediting a neurology textbook; license fees from the Children's Hospital of Philadelphia. All are outside the scope of the current manuscript. Krista Vandenborne declares a research service agreement for the current clinical trial. Outside the current manuscript, she declares grants from the National Institutes of Health and research service support from Sarepta Therapeutics, Catabasis Pharmaceuticals, PTC Therapeutics, Summit Therapeutics, Astellas Pharma, ML Bio/VCU, and Edgewise Therapeutics, all directed to the University of Florida. Sara Cazzaniga, Silvia Montrasio, Federica Alessi, and Paolo Bettica are employees of Italfarmaco SpA, the sponsor of the study. Eugenio Mercuri reports payments for lectures and symposia from Sarepta, PTC, and Roche, and for advisory boards from Sarepta, NS, Santhera, PTC, Roche, Pfizer, wave, Italfarmaco, Dyne, Solid, and Edgewise. All are outside the scope of the current manuscript.

## Supporting information


**Appendix S1:** Supporting information.

## Data Availability

The data that support the findings of this study are available from the corresponding author upon reasonable request.
