# Supplementary material for: Long‐Term Evaluation of Givinostat in Duchenne Muscular Dystrophy, and Natural History Comparisons
Source: Ann Clin Transl Neurol. 2025 Aug 19;12(11):2335–48. doi: 10.1002/acn3.70165 (PMC12623843; doi:10.1002/acn3.70165)
Supplement: Supplementary file 1 — Appendix S1: Supporting information. [file ACN3-12-2335-s001.pdf]

Long-term evaluation of givinostat in Duchenne muscular dystrophy, and natural history comparisons

Craig M McDonald MD, Michela Guglieri MD, Dragana Vučinić MD, Gyula Acsadi MD, PhD, John F Brandsema MD, Claudio Bruno MD, Erika L Finanger MD, Amy Harper MD, Mercedes Lopez Lobato MD, Riccardo Masson MD, Nuria Muelas MD, PhD, Francina Munell MD, PhD, Yoram Nevo MD, Yann Péréon MD, PhD, Han Phan MD, Valeria A Sansone MD, PhD, Mariacristina Scoto MD, PhD, Tracey Willis MD, Richard S Finkel MD, Krista Vandenborne PhD, Sara Cazzaniga MSc, Silvia Montrasio PhD, Federica Alessi MSc, Paolo Bettica MD, PhD, Eugenio Mercuri MD, for the Givinostat Study 51 investigators, the Cooperative International Neuromuscular Research Group (CINRG) DNHS Investigators, and the ImagingDMD investigators.

Supplement

Co-investigators

Name(s)	Investigational site
**Givinostat Study 51 investigators**	
Enrico Bertini, MD	Ospedale Pediatrico Bambino Gesù, Malattie Neuromuscolari e Neurodegenerative, Roma, Italy
Giacomo Pietro Comi, MD	Fondazione IRCCS Ca' Granda Ospedale Maggiore, Policlinico di Milano, SSD Malattie Neuromuscolari e Rare, Milano, Italy
Eugenio Maria Mercuri, MD	Policlinico Universitario Agostino Gemelli, Roma, Italy
Giuseppe Vita, MD	Azienda Ospedaliera Universitaria Policlinico “G. Martino" di Messina, Neurologia e Malattie Neuromuscolari, Messina, Italy
Sonia Messina, MD	
Claudio Bruno, MD	IRCCS Istituto Giannina Gaslini, U.O.S.D. Centro Traslazionale di Miologia e Patologie Neurodegenerative, Genova, Italy
Riccardo Masson, MD	Fondazione IRCCS Istituto Neurologico Carlo Besta, Milan, Italy
Valeria Sansone, MD, PhD	NeuroMuscular Omnicentre (NeMO Clinical Center),, Fondazione Serena Onlus, Pad. n.7 – ASST Grande Ospedale Metropolitano Niguarda, Milan, Italy
Nathalie Goemans, MD	University Hospitals Leuven, Neuromuscular Reference Centre, Child Neurology, Leuven, Brabant, Belgium
Liesbeth De Waele, MD	
Laurent Servais, MD	Hopital de La Citadelle, Centre de Référence des Maladies, Neuromusculaires (CRMN), Liege, Belgium
Teresa Gidaro, MD	Hôpital Armand Trousseau I-Motion - Plateforme d’essais cliniques pédiatriques, Paris, France
Odile Boespflug-Tanguy, MD	
Yann Péréon, MD, PhD	Reference Centre for Neuromuscular Disorders, CHU de Nantes - Hôtel-Dieu, Nantes, France
Jessika Johannsen, MD	Klinik- und Poliklinik fur Kinder- und Jugendmedizin, Universitatsklinikum Hamburg, Hamburg, Germany
Astrid Blaschek, MD	Klinikum der Universitat Munchen, Campus Innenstadt, Munchen, Germany
Ulrike Schara- Schmidt, MD	Universitätsklinikum Essen - Kinder-und Jugendmedizin Neuropadiatrie, Essen, Germany
Erik Niks, MD	Leiden University Medical Center LUMC, Leiden, Netherlands

**Name(s)**	**Investigational site**
Imelda de Groot, MD	Radboud University Medical Centre, Nijmegen, Netherlands
Saskia Houwen-van Opstal, MD	
Andres Nascimento, MD	Neuromuscular Pathology Unit, Hospital Sant Joan de Déu. U.B., Barcelona, Spain
Juan Jesus Vilchez, MD	Neuromuscular Diseases Unit, Neurology Department, Hospital Universitari i Politècnic La Fe, Valencia, Spain and Neuromuscular Reference Centre, ERN-EURO-NMD, Spain.
Nuria Muelas, MD, PhD	
Francina Munell, MD, PhD	Hospital Universitari Vall d'Hebron, Barcelona, Spain
Marcos Madruga Garrido, MD	Hospital Universitario Virgen del Rocio, Sevilla, Spain
Mercedes Lopez Lobato, MD	
Michaela Guglieri, MD	The John Walton Muscular Dystrophy Research Centre, Freeman Hospital, Newcastle University, Institute of Genetic Medicine, Newcastle upon Tyne, United Kingdom
Tracey Willis, MD	The Robert Jones and Agnes Hunt Orthopaedic Hospital NHS Foundation Trust, Oswestry, United Kingdom
Stefan Spinty, MD	Alder Hey Children's Hospital NHS Trust, Liverpool, United Kingdom
Daniel Hawcutt, MD	
Mariacristina Scoto, MD, PhD	Great Ormond Street Hospital & UCL Great Ormond Street Institute of Child Health, Dubowitz Neuromuscular Centre and MRC Centre for NMD, London, United Kingdom
Jean K. Mah, MD	Kinsmen Research Centre, Alberta Children's Hospital, Division of Pediatric Neurology, Cumming School of Medicine, Dept. of Clinical Neurosciences, Calgary, AB, Canada
Laura McAdam, MD	Holland Bloorview Kids Rehabilitation Hospital, Toronto, ON, Canada
Kathryn Selby, MD	The University of British Columbia, Children's and Womens Health Centre of BC Branch, Vancouver, BC, Canada
Katherine Mathews, MD	University of Iowa Children's Hospital, Iowa City, IA, USA
Craig McDonald, MD	University of California, Davis Medical Center, Devis Physical Medicine & Rehabilitation, Sacramento, CA, USA
Craig Zaidman, MD	Washington University School of Medicine in St Louis, Department of Neurology, St. Louis, MO, USA
Barry Byrne, MD	Child Health Research Institute, Department of Pediatrics, Gainesville, FL, USA

**Name(s)**	**Investigational site**
John Brandsema, MD	The Children's Hospital of Philadelphia, Philadelphia, PA, USA
Gyula Acsadi, MD, PhD	Connecticut Children's Medical Center, Division Neurology, Hartford CT, USA
Chamindra Laverty, MD	UC San Diego, Altman Clinical & Translation Research, La Jolla, CA, USA
Amy Harper, MD	Virginia Commonwealth University, Childrens Hospital of Richmond at Virginia Commonwealth University, Richmond, VI, USA
Erika Finanger, MD	Shriners Hospitals for Children – Portland, Portland, OR, USA
Han Phan, MD	Rare Disease Research, LLC, Atlanta, GA, USA
Yoram Nevo, MD	Institute of Neurology – Schneider, Children's Medical Center of Israel, Kaplan, Tel Aviv, Israel
Vedrana Milic Rasic, MD	Clinic of Neurology and Psychiatry for Children and Youth Dr. Belgrade, Serbia
Dragana Vucinic, MD	
**Cooperative International Neuromuscular Research Group (CINRG) Duchenne Natural History Study (DNHS) investigators**	
Craig M. McDonald, MD	University of California, Davis, Sacramento, California, USA
E.K. Henricson, MD	
R.T. Abresch, MD	
N.C. Joyce, MD	
V. Vishwanathan, MD	Sundaram Medical Foundation and Apollo Children's Hospital, Chennai, India
S. Chidambaranathan, MD	
W.D. Biggar, MD	Holland Bloorview Kids Rehabilitation Hospital, Toronto, ON, Canada
Laura C. McAdam, MD	
Jean K. Mah, MD	Alberta Children's Hospital, Calgary, AB, Canada
M. Tulinius, MD	Queen Silvia Children's Hospital, Göteborg, Sweden

Name(s)	Investigational site
A. Cnaan, MD	Children's National Medical Center, Washington DC, USA
L.P. Morgenroth, MD	
R. Leshner, MD	
C. Tesi-Rocha, MD	
M. Thangarajh, MD	
T. Duong, MD	
A. Kornberg, MD	Royal Children's Hospital, Melbourne, Victoria, Australia
M. Ryan, MD	
Yoram Nevo, MD	Hadassah Hebrew University Hospital, Jerusalem, Israel
A. Dubrovsky, MD	Instituto de Neurociencias Fundacion Favaloro, Buenos Aires, Argentina
P.R. Clemens, MD	University of Pittsburgh and Children's Hospital of Pittsburgh, Pittsburgh, PA, USA
H. Abdel-Hamid, MD	
A.M. Connolly, MD	Washington University in St Louis, St Louis, MO, USA
A. Pestronk, MD	
J. Teasley, MD	Children's Hospital of Virginia, Richmond, VA, USA
T.E. Bertorini, MD	University of Tennessee, Memphis, TN, USA
R. Webster, MD	Children's Hospital at Westmead, Sydney, New South Wales
H. Kolski, MD	University of Alberta, Edmonton, AB, Canada
N. Kuntz, MD	Mayo Clinic, Rochester, MO, USA
S. Driscoll, MD	
J.B. Bodensteiner, MD	
J. Carlo, MD	University of Puerto Rico, San Juan, Puerto Rico
K. Gorni, MD	University of Pavia and Niguarda Ca' Granda Hospital, Milan, Italy
T. Lotze, MD	Texas Children's Hospital, Houston, TX, USA

Name(s)	Investigational site
J.W. Day, MD	University of Minnesota, Minneapolis, MO, USA
P. Karachunski, MD	
**ImagingDMD investigators**	
Krista Vandenborne, PhD	University of Florida, Gainesville, Florida, USA
Alison M Barnard, PT, PhD	
Rebecca J Willcocks, PhD	
Donavon J Lott PT, PhD	
Sean C Forbes, PhD	
Glenn A Walter, PhD	
Erika L Finanger, MD	Oregon Health and Science University, Portland, OR, USA
William D Rooney	
Gihan Tennekoon MD	The Children’s Hospital, Philadelphia, PA, USA
John Brandsema, MD	
Ann T Harrington, PT, PhD	

Supplementary methods

Givinostat dose

Supplementary Table 1. Givinostat dose.

	Weight (kg)								
	≥10 and <12.5	≥12.5 and <20	≥20 and <25	≥25 and <30	≥30 and <40	≥40 and <50	≥50 and <60	≥60 and <70	≥70
**Treatment-naïve patients, starting dose**									
Starting dose (mg) bid	13.3	16.7	20.0	23.3	26.7	33.3	36.7	40.0	46.7
Oral suspension (mL) bid	1.3	1.7	2.0	2.3	2.7	3.3	3.7	4.0	4.7
**Patients entering the extension study who received givinostat 37.5 mg bid in the Phase 2 study**									
Starting dose (mg) bid	NA	NA	NA	NA	40.0	50.0	55.0	60.0	70.0
Oral suspension (mL) bid	NA	NA	NA	NA	4.0	5.0	5.5	6.0	7.0
**Patients entering the extension study who received givinostat 25 mg bid in the Phase 2 study**									
Starting dose (mg) bid	NA	NA	NA	NA	27.0	33.0	37.0	40.0	47.0
Oral suspension (mL) bid	NA	NA	NA	NA	2.7	3.3	3.7	4.0	4.7
**Patients entering the extension study who received givinostat bid in the Phase 3 study**									
**Dose level A**									
Dose at end of prior study (mg) bid	20	25	30	35	40	50	55	60	70
Oral suspension (mL) bid	2.0	2.5	3.0	3.5	4.0	5.0	5.5	6.0	7.0

	Weight (kg)								
	≥10 and <12.5	≥12.5 and <20	≥20 and <25	≥25 and <30	≥30 and <40	≥40 and <50	≥50 and <60	≥60 and <70	≥70
**Dose level B**									
Dose at end of prior study (mg) bid	13.3	16.7	20.0	23.3	26.7	33.3	36.7	40.0	46.7
Oral suspension (mL) bid	1.3	1.7	2.0	2.3	2.7	3.3	3.7	4.0	4.7
**Dose level C**									
Dose at end of prior study (mg) bid	10.6	13.4	16.0	18.6	21.4	26.6	29.4	32.0	37.4
Oral suspension (mL) bid	1.1	1.3	1.6	1.9	2.1	2.7	2.9	3.2	3.7

bid, twice daily; NA, not applicable.

Givinostat dose increases

The givinostat dose was to be increased if all of the following occurred:

- Weight gain (at baseline, and then yearly)*
- Platelets >120 x 10^9/L in the previous 4 months of treatment;
- No severe drug-related adverse events occurred;
- No drug-related serious adverse event occurred.

*If the dose was increased for weight gain, a complete blood count test was to be performed at least 2 weeks after the dose increase.

Givinostat safety stopping rules

Study drug was to be permanently interrupted if any of the following occurred:

- severe drug-related diarrhoea (i.e., increase of ≥7 stools per day);
- any drug-related serious adverse event;
- QTcF >500 msec;
- platelet count ≤50 x 10^9/L;

- white blood cells ≤2.0 x 10^9/L;
- hemoglobin ≤8.0 g/dL.

Study drug was to be temporarily stopped if any of the following occurred:

- moderate or severe diarrhoea (i.e., increase more than 4 stools per day);
- platelet count <75 x 10^9/L but >50 x 10^9/L;
- white blood cell <3.0 x 10^9/L but >2.0 x 10^9/L;
- hemoglobin <10.0 g/dL but >8.0 g/dL;
- triglycerides >300 mg/dL (3.42 mmol/L) in fasting condition.

If the dose level when givinostat was stopped was Dose Level A, givinostat could be resumed at a dose reduced by 1/3 (Dose Level B, see Supplementary Table 1), once platelet count was >150 x 10^9/L and/or white blood cell and/or hemoglobin were normalized, and/or triglycerides returned to <300 mg/dL (3.42 mmol/L), and/or diarrhoea was mild. If the dose level when givinostat was stopped was Dose Level B, givinostat could be resumed at a dose reduced by 20% (Dose Level C), once these criteria were met. If the dose level when givinostat was stopped was Dose Level C, givinostat could be resumed at the same dose, once these criteria were met. After givinostat was recommenced, a complete blood count was to be performed every 2 weeks for 8 weeks. After 8 weeks, providing the platelet count was >120 x 10^9/L and/or white blood cell was ≥3 x 10^9/L and/or hemoglobin was ≥10.0 mg/dL, patients could continue the study as per scheduled visits.

Inclusion criteria

1. Must have participated in one of the prior givinostat studies in DMD and have attended the end of study visit

or

must have been screened in the Phase 3 study and met:

- all the inclusion criteria and none of the exclusion criteria,
- had a baseline vastus lateralis muscle fat fraction (VL MFF) assessed by MRS in the range ≤5% or >30%, i.e. included in Group B,
- never been randomized because enrolment into Group B was completed;

2. Aged ≥6 years old;

3. Able to give informed assent and/or consent in writing signed by the subject and/or parent/legal guardian (according to local regulations);

4. Willing to use adequate contraception:

- Contraceptive methods must be used since the prior study until 3 months after the last dose of study drug, including the following:
  - True abstinence (absence of any sexual intercourse), when in line with the preferred and usual lifestyle of the subject. Periodic abstinence (e.g. calendar, ovulation, symptothermal, postovulation methods) and withdrawal were not acceptable methods of contraception.
  - Condom with spermicide and the female partner must use an acceptable method of contraception, such as an oral, transdermal, injectable or implanted steroid-based contraceptive, or a diaphragm or a barrier method of contraception in conjunction with spermicidal jelly such as for example cervical cap with spermicide jelly.

Exclusion criteria

1. Use of any pharmacologic treatment, other than corticosteroids, that might have had an effect on muscle strength or function within 3 months prior to enrolment (e.g., growth hormone); Vitamin D, calcium, and any other supplements were allowed;

2. Use of any current investigational drug other than givinostat;

3. Presence of other clinically significant disease, which, in the Investigator's opinion, could have adversely affected the safety of the subject, making it unlikely that the course of treatment or follow-up would be completed, or that could have impaired the assessment of study results;

4. Diagnosis of other uncontrolled neurological diseases or presence of relevant uncontrolled somatic disorders that are not related to DMD;

5. Platelets, white blood cells or hemoglobin at screening <lower limit of normal;

6. Triglycerides >300 mg/dL (3.42 mmol/L) in fasting condition at screening;

7. Inadequate renal function, as defined by serum cystatin C >2 x the upper limit of normal (ULN) at screening;

8. Heart failure (New York Heart Association Class III or IV);

9. Current liver disease or impairment, including but not limited to an elevated total bilirubin (>1.5 x ULN), unless secondary to Gilbert's disease or pattern consistent with Gilbert's;

10. Baseline QTcF >450 msec, or history of additional risk factors for torsades de pointes (e.g., heart failure, hypokalemia, or family history of long QT syndrome);

11. Psychiatric illness/social situation rendering the subject unable to understand and comply with the muscle function tests and/or with the study protocol procedures.

12. Any hypersensitivity to the components of study medication;

13. Sorbitol intolerance or sorbitol malabsorption or have the hereditary form of fructose intolerance.

Protocol amendments

At the time of the database lock, the protocol had been amended three times. The main amendments were the addition of triglyceride and renal function exclusion criteria (see supplement; criteria 6 and 7), the inclusion of a triglyceride criterion to the temporary stopping rules (see supplement), modification of the platelet count threshold in the safety rules (from >150x10^9/L to >120x10^9/L) and the addition of the SF-36 as quality-of-life questionnaire for those participants who become adults during the open-label extension study. In addition, the following country-specific protocol amendments were implemented:

- Germany: The maximum open-label extension study duration was limited to 2 years, requiring the submission of a protocol amendment with data collected every 2 years, in order to continue the trial until approval.
- France: The minimum age for inclusion was set to 7 years and 6 months.

Handling of missing data for the open-label extension study

For the efficacy analyses, any missing data for continuous values for a subject were classified as follows:

1. The patient had missing data because of reasons other than being non-ambulatory or physically unable to perform the test/assessment

2. The patient had missing data because of being either non-ambulatory or otherwise physically unable to perform the test/assessment.

Any missing values classified as 1 were imputed using the mean (or the mode, depending on the nature of the variable) of all non-missing values for the respective measurement and

time point across all relevant (i.e., ambulant/non-ambulant depending on the assessment) subjects in the respective treatment group (givinostat, delayed givinostat or naïve givinostat). Any missing values classified as 2 were imputed by setting the value to zero or to twice the maximum non-missing value recorded across all subjects depending on the directionality of the test.

Patients losing ambulation after baseline had results subsequent to the loss of ambulation imputed to worst case (Class 2). Note that missing data at any completely missing visits prior to withdrawal or completing the study or missing data where the visit was completed but the data was missing with missing reason for not attempting the assessment were imputed as follows: where the final assessment prior to skipped assessment was missing and determined to be Class 2 missing data, all missing assessments between the performed visits were also be considered Class 2. Otherwise, these were considered Class 1.

The following variables had Class 2 missing data which represented higher values as worse outcome:

- 4 stair climb
- Time to rise from the floor
- Time to run/walk 10 meters
- Egen Klassifikation.

The following variables had Class 2 missing data which represented lower values as worse outcome:

- Performance Upper Limb
- Motor Function Measure
- North Star Ambulatory Assessment
- 6-min walking test
- Muscle strength test – handheld myometry
- Barthel Index
- Pediatric Quality of Life Inventory (Class 1 only).

Endpoint definitions for comparisons with natural history datasets

Loss of rise from floor was defined as the patient being unable to perform the rise from floor test due to physical inability. Similarly, loss of ability to perform 4SC was defined as the patient being unable to perform the 4 standard stairs climb due to physical inability.

Loss of ambulation was defined as satisfying both of the following criteria at the same visit: unable to perform the 6MWT due to physical inability; and unable to complete the 10MWT in 30 seconds or less without any support or devices. As the 10MWT grading data were not collected in the CINRG, loss of ambulation was defined as ambulatory status of 2 (i.e. non-ambulatory).

Results

Safety

Supplementary Table 2. Platelet, triglyceride and example echocardiography values.

Parameter	Received givinostat throughout*	Received placebo in prior study*	Not included in prior study*
**Minimum platelet count**			
All patients			
Baseline value, 10^9/L, mean (SD)	246.7 (83.57) (N=102)	315.5 (78.42) (N=50)	332.4 (80.73) (N=28)
Minimum value during treatment period, 10^9/L, mean (SD)	180.4 (45.48) (N=110)	159.8 (58.82) (N=54)	186.6 (53.29) (N=30)
Time to minimum, days, mean (SD)	258.1 (339.02) (N=110)	144.8 (162.73) (N=54)	84.2 (91.81) (N=30)
Patients who reduced dose due to platelet count decrease	(N=5)	(N=4)	(N=1)
Minimum value during treatment period, 10^9/L, mean (SD)	122.2 (20.73)	113.3 (18.03)	94.0
Time to minimum, days, mean (SD) [range]	448.4 (280.87) [4, 672]	73.5 (48.05) [14, 113]	21.0
Time to recovery, days, mean (SD) [range]	34.0 (38.66) [9, 91] (N=4)	247.7 (361.46) [34, 665] (N=3)	(N=0)
**Maximum triglyceride count**			
All patients			
Baseline value, mmol/L, mean (SD)	1.806 (0.7960) (N=104)	1.505 (0.6888) (N=52)	1.520 (0.6023) (N=30)
Maximum value during treatment period, mmol/L, mean (SD)	2.689 (1.0780) (N=109)	2.773 (1.2721) (N=53)	2.600 (1.0994) (N=30)
Time to maximum, days, mean (SD)	252.4 (298.20) (N=109)	195.4 (219.87) (N=53)	140.9 (128.82) (N=30)
Patients who reduced dose due to triglyceride increase	(N=2)	(N=1)	(N=0)
Maximum value during treatment period, mmol/L, mean (SD)	4.940 (0.4808)	3.880	–

Parameter	Received givinostat throughout*	Received placebo in prior study*	Not included in prior study*
Time to maximum, days, mean (SD) [range]	71.5 (58.69) [30, 113]	562	–
Time to recovery, days	1019 (N=1)	(N=0)	–
**Left ventricular ejection fraction, %**			
Baseline, mean (SD)	62.174 (6.7357) (N=88)	60.423 (7.7345) (N=45)	63.556 (5.9800) (N=26)
Change from baseline at Month 12, mean (SD)	–1.520 (8.9157) (N=45)	1.217 (10.0823) (N=22)	1.047 (8.6603) (N=22)
Change from baseline at Month 24, mean (SD)	–2.023 (8.2256) (N=26)	3.606 (6.8832) (N=10)	–
**Left ventricular end diastolic diameter internal dimension, mm**			
Baseline, mean (SD)	39.994 (4.0793) (N=101)	40.628 (5.6539) (N=50)	40.040 (3.8635) (N=30)
Change from baseline at Month 12, mean (SD)	1.377 (3.3912) (N=56)	–0.064 (3.6571) (N=28)	–0.029 (3.6076) (N=26)
Change from baseline at Month 24, mean (SD)	1.218 (4.2434) (N=36)	–2.182 (2.9718) (N=11)	–

*All patients were also receiving systemic corticosteroids for the full duration of the follow-up period.

Supplementary Figure 1. Mean observed values for creatinine.

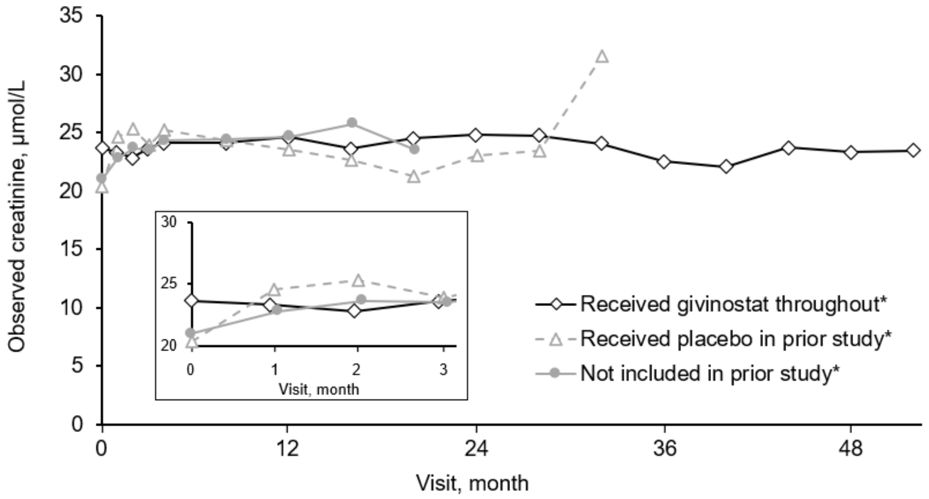

N=					
Givinostat throughout	103	73	39	14	16
Placebo in prior study	52	37	16	†	†
Not included	30	25	†	†	†

*All patients were also receiving systemic corticosteroids for the full duration of the follow-up period. †No patients have reached this timepoint. N indicates the number available for assessment.

Supplementary Figure 2. Mean observed values for cystatin C.

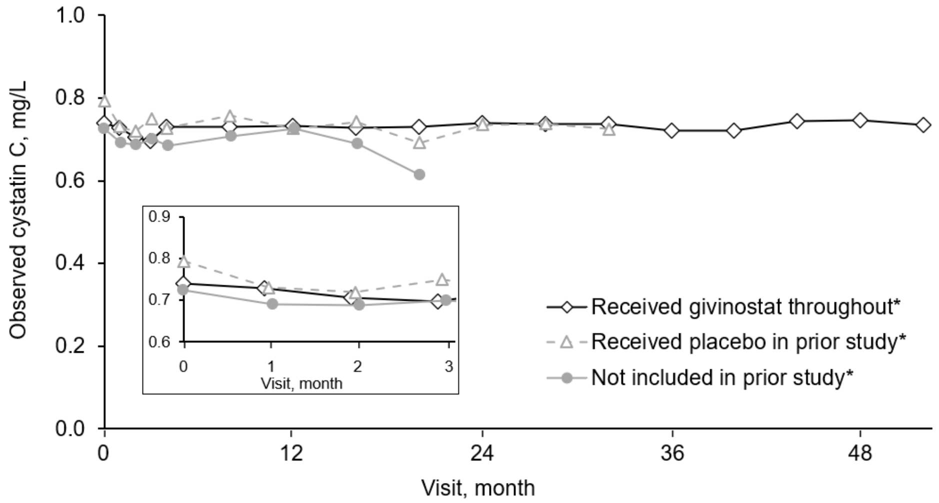

N=					
Givinostat throughout	93	69	38	14	16
Placebo in prior study	40	37	15	†	†
Not included	30	24	†	†	†

*All patients were also receiving systemic corticosteroids for the full duration of the follow-up period. †No patients have reached this timepoint. N indicates the number available for assessment.

Efficacy

Supplementary Table 3. Exploratory comparison of efficacy endpoints at 24 months, between patients who received givinostat vs. placebo in the prior studies.

Endpoint	Received givinostat throughout	Received placebo in prior study
**Ambulant patients**	**(N=38)**	**(N=17)**
4-stair climb velocity, tasks.sec^{-1}	–0.33 (–0.440, –0.217)	–0.35 (–0.515, –0.187)
Comparison	0.02 (–0.177, 0.222)	
4-stair climb, sec	2.22 (1.679, 2.948)	2.14 (1.410, 3.258)
Comparison	1.04 (0.626, 1.723)	
10 m velocity, m.sec^{-1}	–0.57 (–0.722, –0.423)	–0.64 (–0.858, –0.417)
Comparison	0.06 (–0.203, 0.333)	
Time to walk 10 m, sec	104.17 (48.797, 159.535)	112.97 (30.827, 195.110)
Comparison	–8.80 (–108.135, 90.530)	
Rise from floor velocity, sec^{-1}	–0.03 (–0.049, –0.014)	–0.04 (–0.070, –0.018)
Comparison	0.01 (–0.019, 0.044)	
Time to rise from floor, sec	36.44 (18.257, 54.622)	43.89 (17.009, 70.779)
Comparison	–7.45 (–40.115, 25.206)	
NSAA	–7.77 (–9.749, –5.790)	–6.12 (–9.034, –3.206)
Comparison	–1.65 (–5.181, 1.882)	
6MWT, m	–118.28 (–152.327, –84.226)	–104.70 (–154.891, –54.511)
Comparison	–13.58 (–74.388, 47.237)	
Left knee extension, N	–8.04 (–13.990, –2.085)	–9.23 (–18.018, –0.441)
Comparison	1.19 (–9.471, 11.855)	
Right knee extension, N	–10.99 (–16.785, –5.203)	–9.22 (–17.745, –0.688)
Comparison	–1.78 (–12.130, 8.574)	
Left elbow flexion, N	–4.59 (–8.443, –0.739)	1.91 (–3.815, 7.635)
Comparison	–6.50 (–13.445, 0.443)	
Right elbow flexion, N	–5.11 (–8.524, –1.689)	0.53 (–4.525, 5.591)
Comparison	–5.64 (–11.785, 0.506)	

Endpoint	Received givinostat throughout	Received placebo in prior study
**All patients**	**(N=43)**	**(N=18)**
PUL total score, N	–3.25 (–5.406, –1.090)	–2.18 (–5.509, 1.151)
Comparison	–1.07 (–5.052, 2.915)	
PUL high-level shoulder dimension, N	–2.11 (–3.099, –1.121)	–1.66 (–3.177, –0.142)
Comparison	–0.45 (–2.265, 1.366)	
PUL mid-level elbow dimension, N	–1.23 (–2.128, –0.340)	–0.44 (–1.802, 0.929)
Comparison	–0.80 (–2.434, 0.838)	
PUL distal wrist and hand dimension, N	–0.18 (–0.740, 0.370)	–0.07 (–0.919, 0.772)
Comparison	–0.11 (–1.131, 0.910)	
MFM scale total score	–7.64 (–10.494, –4.778)	–7.96 (–12.203, –3.718)
Comparison	0.32 (–4.823, 5.472)	
MFM scale standing and transfers score	–6.25 (–8.341, –4.159)	–6.48 (–9.618, –3.333)
Comparison	0.23 (–3.568, 4.019)	
MFM scale axial and proximal motor function score	–1.41 (–2.451, –0.360)	–1.42 (–2.963, 0.129)
Comparison	0.01 (–1.863, 1.886)	
MFM scale distal motor function score	0.16 (–0.106, 0.433)	–0.06 (–0.465, 0.335)
Comparison	0.23 (–0.263, 0.720)	
Forced vital capacity, % predicted	–11.70 (–16.828, –6.567) (N=38)	–10.71 (–18.841, –2.579) (N=14)
Comparison	–0.99 (–10.715, 8.739)	
Peak expiratory flow, % predicted	–1.71 (–8.491, 5.078) (N=39)	–5.98 (–17.030, 5.078) (N=14)
Comparison	4.27 (–8.810, 17.349)	

Treatment group data are least squares mean change from baseline (95% confidence interval), with givinostat throughout vs. placebo in prior study comparisons least squares means (95% confidence interval), except 4-stair climb, for which treatment group data are geometric least squares means (95% confidence interval), and the comparison is geometric least squares mean ratio (95% confidence interval). NSAA, North Star Ambulatory Assessment; 6MWT, 6 min walk test; PUL, Performance of the Upper Limb; MFM, Motor Function Measure.

Ambulant patients

Supplementary Figure 3. Change from baseline in rise from floor velocity (ambulant patients).

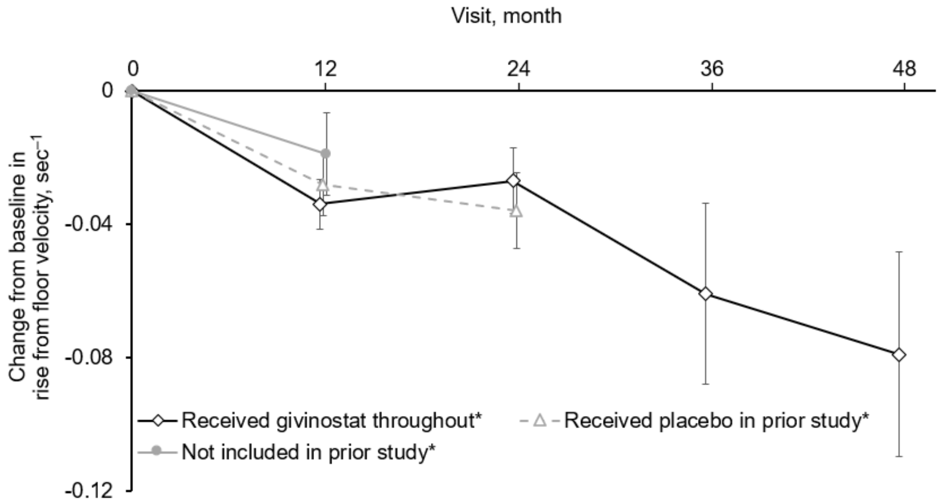

N=					
Givinostat throughout	102	66	38	11	12
Placebo in prior study	51	35	17	†	†
Not included	30	27	†	†	†

*All patients were also receiving systemic corticosteroids for the full duration of the follow-up period. †No patients have reached this timepoint. Data are mean and standard error, with N indicating the number available for assessment. Mean (SD) values at baseline were 0.136 (0.0958), 0.124 (0.0812), and 0.204 (0.0685) sec^{-1} in the givinostat throughout, prior placebo, and not included groups, respectively.

Supplementary Figure 4. Change from baseline in time to rise from floor (ambulant patients).

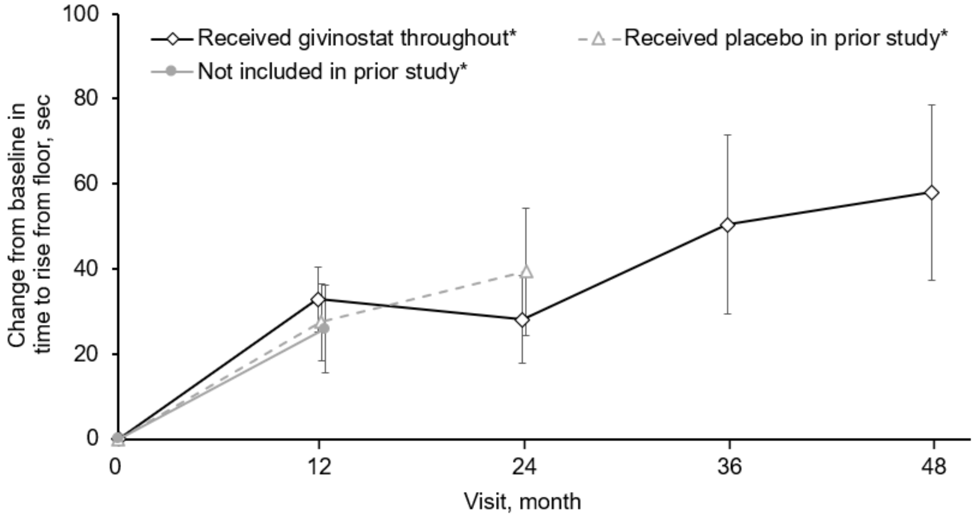

N=					
Givinostat throughout	102	66	38	11	12
Placebo in prior study	51	35	17	†	†
Not included	30	27	†	†	†

*All patients were also receiving systemic corticosteroids for the full duration of the follow-up period. †No patients have reached this timepoint. Data are mean and standard error, with N indicating the number available for assessment. Mean (SD) values at baseline were 39.631 (58.7373), 34.841 (54.9647), and 5.566 (2.0607) sec in the givinostat throughout, prior placebo, and not included groups, respectively.

Supplementary Figure 5. Change from baseline in 4 stair climb (4SC) velocity (ambulant patients).

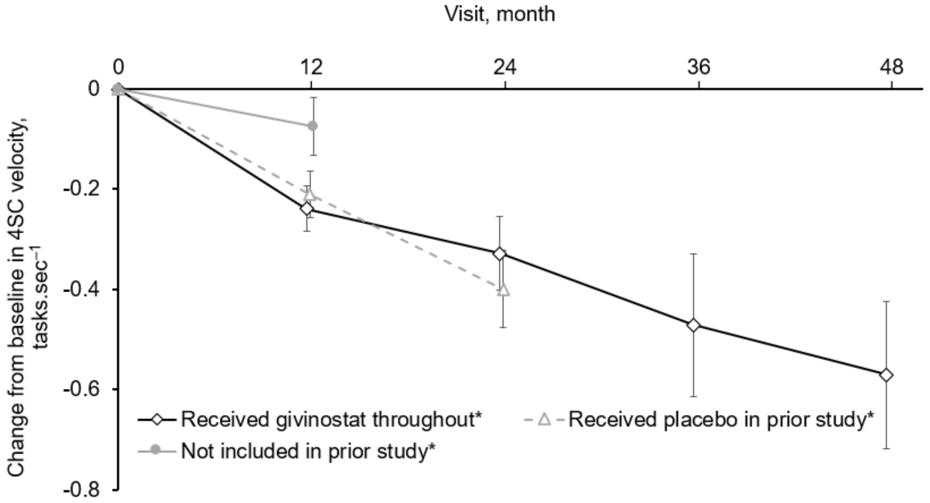

N=					
Givinostat throughout	102	66	38	12	12
Placebo in prior study	51	35	17	†	†
Not included	30	27	†	†	†

*All patients were also receiving systemic corticosteroids for the full duration of the follow-up period. †No patients have reached this timepoint. Data are mean and standard error, with N indicating the number available for assessment. Mean (SD) values at baseline were 1.035 (0.5445), 0.965 (0.4824), and 1.256 (0.4325) tasks.sec^{-1} in the givinostat throughout, prior placebo, and not included groups, respectively.

Supplementary Figure 6. Change from baseline in 4 stair climb (4SC) (ambulant patients).

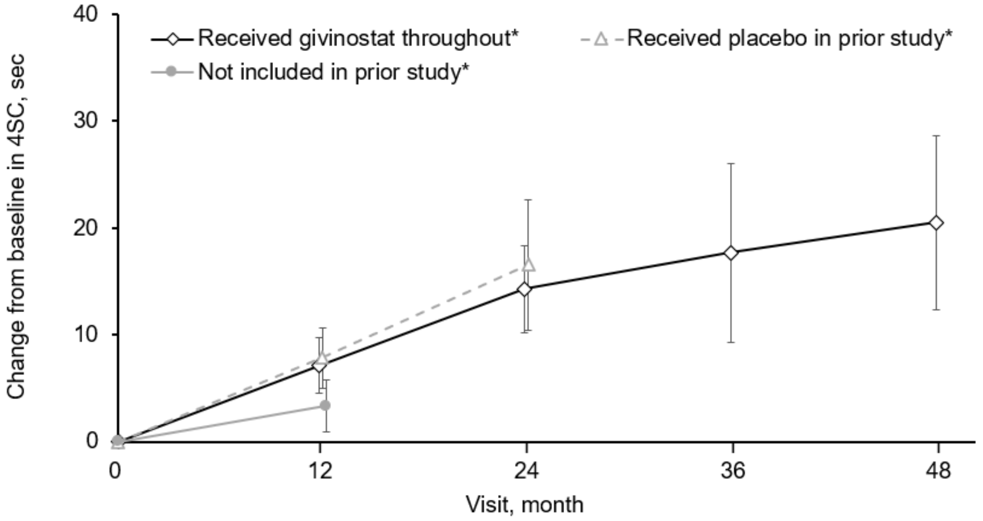

N=					
Givinostat throughout	102	66	38	12	12
Placebo in prior study	51	35	17	†	†
Not included	30	27	†	†	†

*All patients were also receiving systemic corticosteroids for the full duration of the follow-up period. †No patients have reached this timepoint. Data are mean and standard error, with N indicating the number available for assessment. Mean (SD) values at baseline were 7.969 (14.5775), 6.758 (10.0173), and 3.579 (1.2751) sec in the givinostat throughout, prior placebo, and not included groups, respectively.

Supplementary Figure 7. Change from baseline in 10 m walk velocity (ambulant patients).

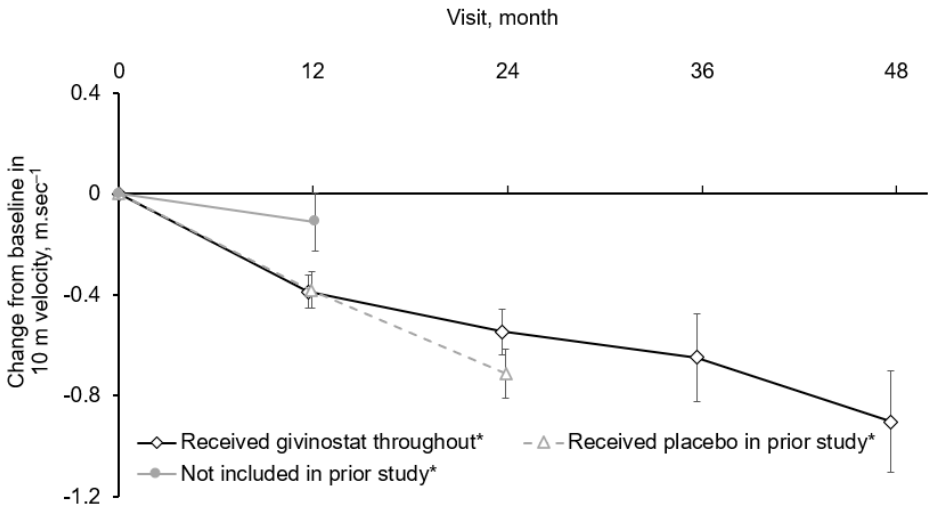

N=					
Givinostat throughout	102	66	38	12	12
Placebo in prior study	51	35	17	†	†
Not included	30	27	†	†	†

*All patients were also receiving systemic corticosteroids for the full duration of the follow-up period. †No patients have reached this timepoint. Data are mean and standard error, with N indicating the number available for assessment. Mean (SD) values at baseline were 1.677 (0.6041), 1.657 (0.4460), and 1.905 (0.5855) m.sec^{-1} in the givinostat throughout, prior placebo, and not included groups, respectively.

Supplementary Figure 8. Change from baseline in 10 m walk (ambulant patients).

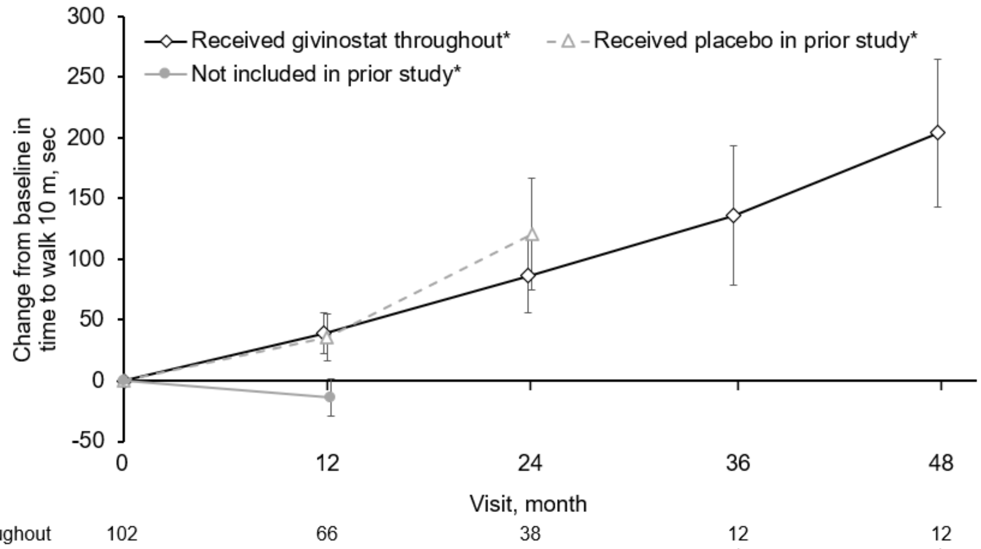

N=					
Givinostat throughout	102	66	38	12	12
Placebo in prior study	51	35	17	†	†
Not included	30	27	†	†	†

*All patients were also receiving systemic corticosteroids for the full duration of the follow-up period. †No patients have reached this timepoint. Data are mean and standard error, with N indicating the number available for assessment. Mean (SD) values at baseline were 18.425 (69.2360), 6.539 (2.0599), and 18.980 (74.6176) sec in the givinostat throughout, prior placebo, and not included groups, respectively.

Supplementary Figure 9. Change from baseline in North Star Ambulatory Assessment (NSAA) total score (ambulant patients).

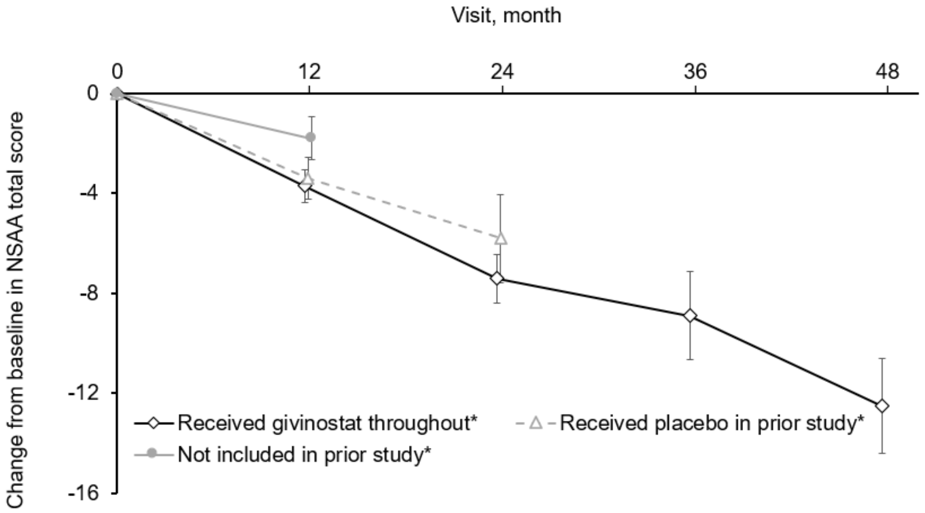

N=					
Givinostat throughout	102	66	38	12	12
Placebo in prior study	51	35	17	†	†
Not included	30	27	†	†	†

*All patients were also receiving systemic corticosteroids for the full duration of the follow-up period. †No patients have reached this timepoint. Data are mean and standard error, with N indicating the number available for assessment. Mean (SD) values at baseline were 20.3 (7.08), 19.9 (6.52), and 23.6 (6.16) in the givinostat throughout, prior placebo, and not included groups, respectively.

Supplementary Figure 10. Change from baseline in 6 min walk test (6MWT) (ambulant patients).

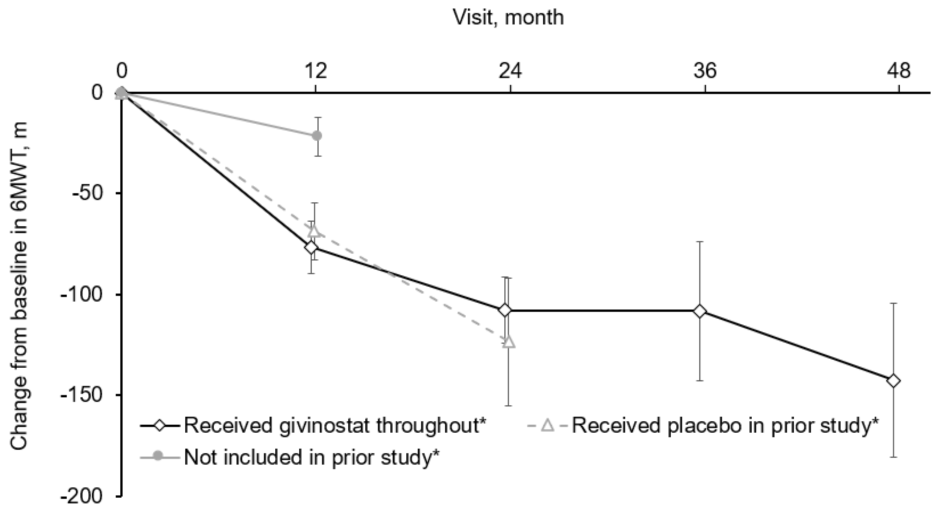

N=					
Givinostat throughout	102	66	38	12	12
Placebo in prior study	51	35	17	†	†
Not included	30	27	†	†	†

*All patients were also receiving systemic corticosteroids for the full duration of the follow-up period. †No patients have reached this timepoint. Data are mean and standard error, with N indicating the number available for assessment. Mean (SD) values at baseline were 350.329 (104.7516), 347.673 (83.8204), and 386.814 (71.8823) m in the givinostat throughout, prior placebo, and not included groups, respectively.

Supplementary Figure 11. Change from baseline in left knee extension (ambulant patients).

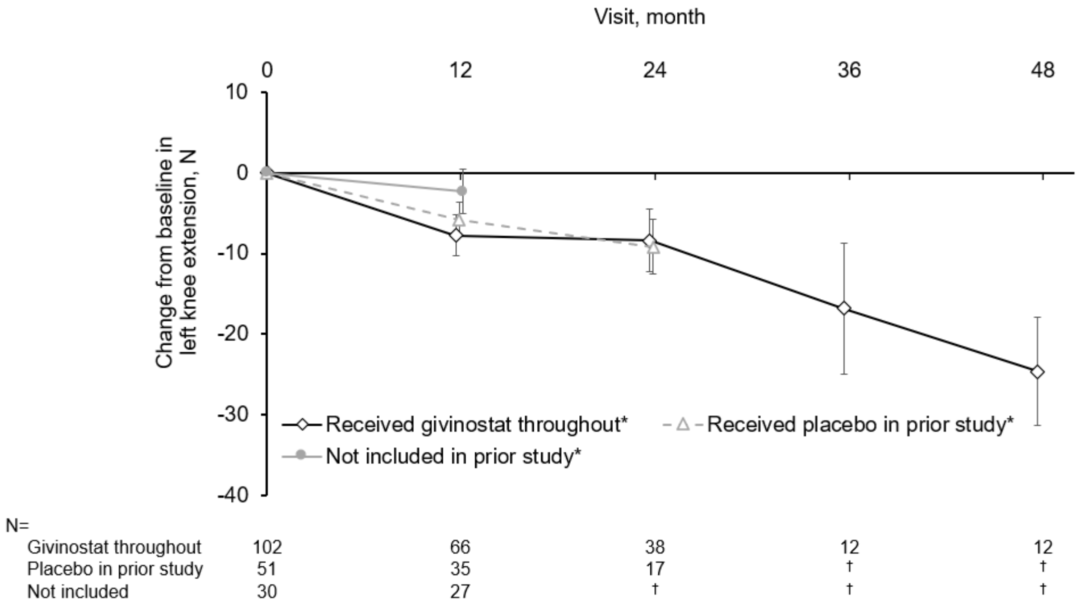

*All patients were also receiving systemic corticosteroids for the full duration of the follow-up period. †No patients have reached this timepoint. Data are mean and standard error, with N indicating the number available for assessment. Mean (SD) values at baseline were 45.43 (19.944), 44.27 (23.683), and 56.14 (20.484) N in the givinostat throughout, prior placebo, and not included groups, respectively.

Supplementary Figure 12. Change from baseline in right knee extension (ambulant patients).

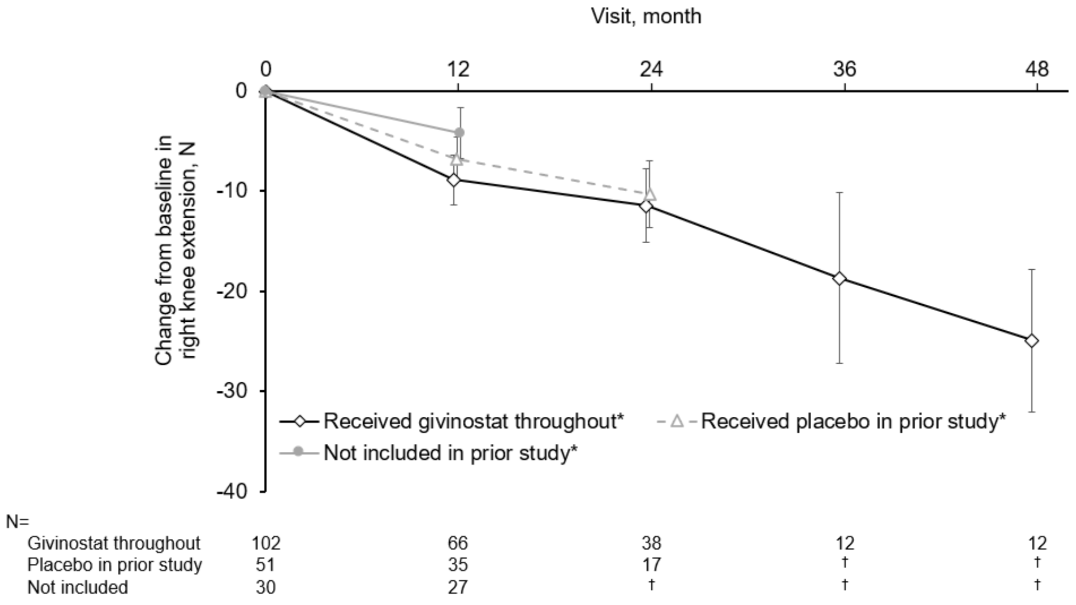

*All patients were also receiving systemic corticosteroids for the full duration of the follow-up period. †No patients have reached this timepoint. Data are mean and standard error, with N indicating the number available for assessment. Mean (SD) values at baseline were 47.99 (20.731), 47.37 (25.486), and 60.34 (22.161) N in the givinostat throughout, prior placebo, and not included groups, respectively.

Supplementary Figure 13. Change from baseline in left elbow flexion (ambulant patients).

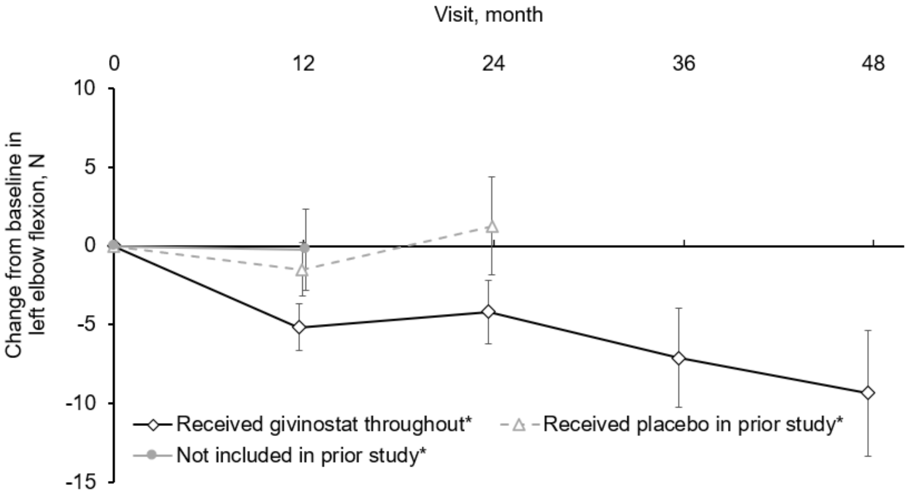

N=	0	12	24	36	48
Givinostat throughout	102	66	38	12	12
Placebo in prior study	51	35	17	†	†
Not included	30	27	†	†	†

*All patients were also receiving systemic corticosteroids for the full duration of the follow-up period. †No patients have reached this timepoint. Data are mean and standard error, with N indicating the number available for assessment. Mean (SD) values at baseline were 34.74 (12.194), 38.03 (17.521), and 39.98 (11.827) N in the givinostat throughout, prior placebo, and not included groups, respectively.

Supplementary Figure 14. Change from baseline in right elbow flexion (ambulant patients).

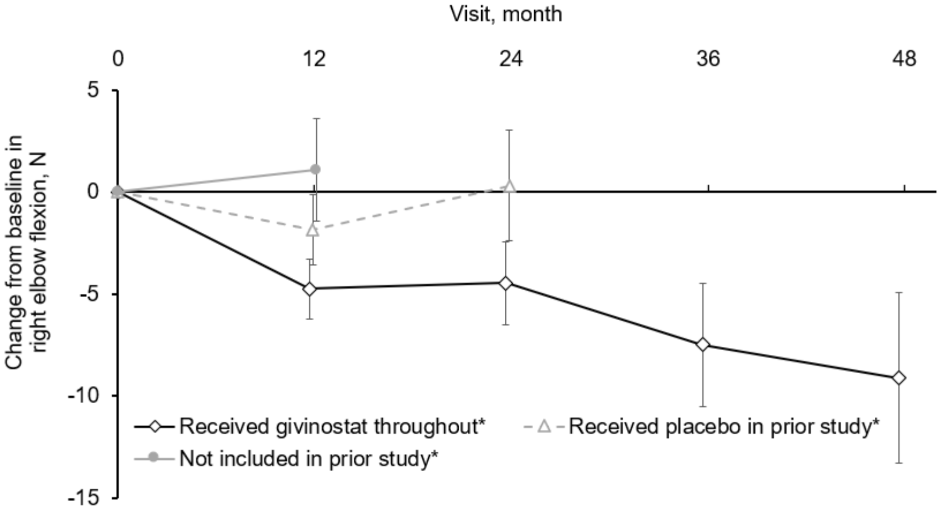

N=	0	12	24	36	48
Givinostat throughout	102	66	38	12	12
Placebo in prior study	51	35	17	†	†
Not included	30	27	†	†	†

*All patients were also receiving systemic corticosteroids for the full duration of the follow-up period. †No patients have reached this timepoint. Data are mean and standard error, with N indicating the number available for assessment. Mean (SD) values at baseline were 35.10 (12.759), 39.07 (18.622), and 40.43 (11.858) N in the givinostat throughout, prior placebo, and not included groups, respectively.

All patients

Supplementary Figure 15. Change from baseline in Performance of the Upper Limb (PUL) total score (overall population).

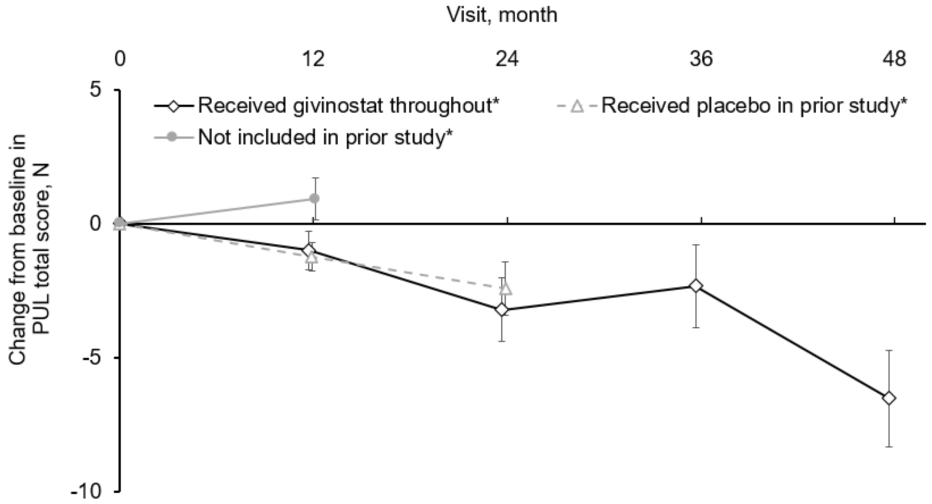

N=					
Givinostat throughout	110	73	43	16	16
Placebo in prior study	54	37	18	†	†
Not included	30	27	†	†	†

*All patients were also receiving systemic corticosteroids for the full duration of the follow-up period. †No patients have reached this timepoint. Data are mean and standard error, with N indicating the number available for assessment. Mean (SD) values at baseline were 37.498 (5.0441), 38.392 (3.1086), and 38.933 (4.1683) N in the givinostat throughout, prior placebo, and not included groups, respectively.

Supplementary Figure 16. Change from baseline in Performance of the Upper Limb (PUL) high-level shoulder dimension (overall population).

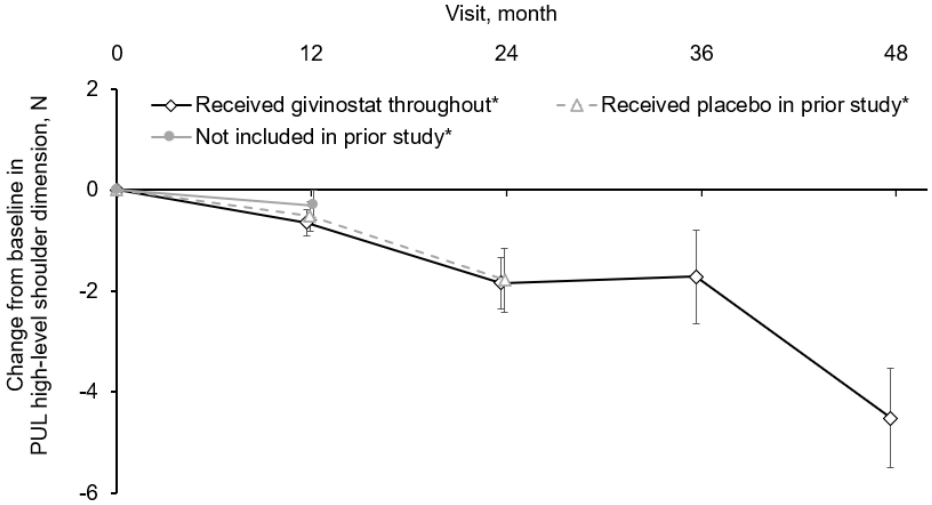

N=					
Givinostat throughout	110	73	43	16	16
Placebo in prior study	54	37	18	†	†
Not included	30	27	†	†	†

*All patients were also receiving systemic corticosteroids for the full duration of the follow-up period. †No patients have reached this timepoint. Data are mean and standard error, with N indicating the number available for assessment. Mean (SD) values at baseline were 9.957 (1.9057), 10.392 (1.5910), and 11.200 (0.9248) N in the givinostat throughout, prior placebo, and not included groups, respectively.

Supplementary Figure 17. Change from baseline in Performance of the Upper Limb (PUL) mid-level elbow dimension (overall population).

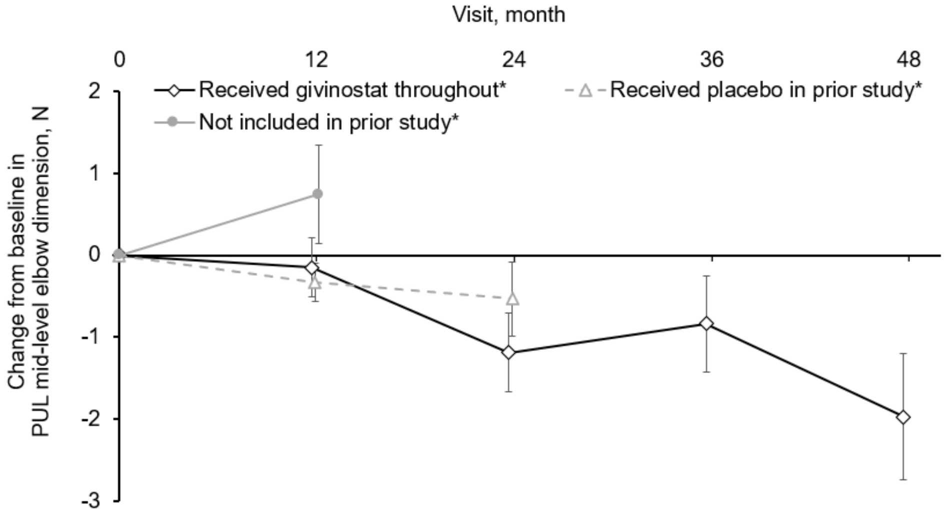

N=					
Givinostat throughout	110	73	43	16	16
Placebo in prior study	54	37	18	†	†
Not included	30	27	†	†	†

*All patients were also receiving systemic corticosteroids for the full duration of the follow-up period. †No patients have reached this timepoint. Data are mean and standard error, with N indicating the number available for assessment. Mean (SD) values at baseline were 15.875 (2.4885), 16.059 (1.3376), and 16.100 (3.1112) N in the givinostat throughout, prior placebo, and not included groups, respectively.

Supplementary Figure 18. Change from baseline in Performance of the Upper Limb (PUL) distal wrist and hand dimension (overall population).

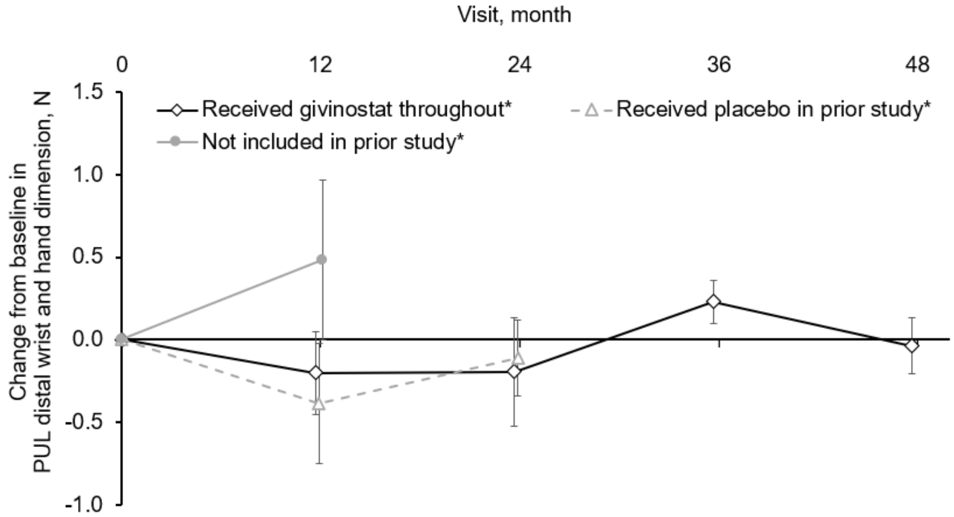

N=					
Givinostat throughout	110	73	43	16	16
Placebo in prior study	54	37	18	†	†
Not included	30	27	†	†	†

*All patients were also receiving systemic corticosteroids for the full duration of the follow-up period. †No patients have reached this timepoint. Data are mean and standard error, with N indicating the number available for assessment. Mean (SD) values at baseline were 11.666 (1.4208), 11.941 (0.9598), and 11.633 (2.3851) N in the givinostat throughout, prior placebo, and not included groups, respectively.

Supplementary Figure 19. Change from baseline in Motor Function Measure (MFM) scale total score (overall population).

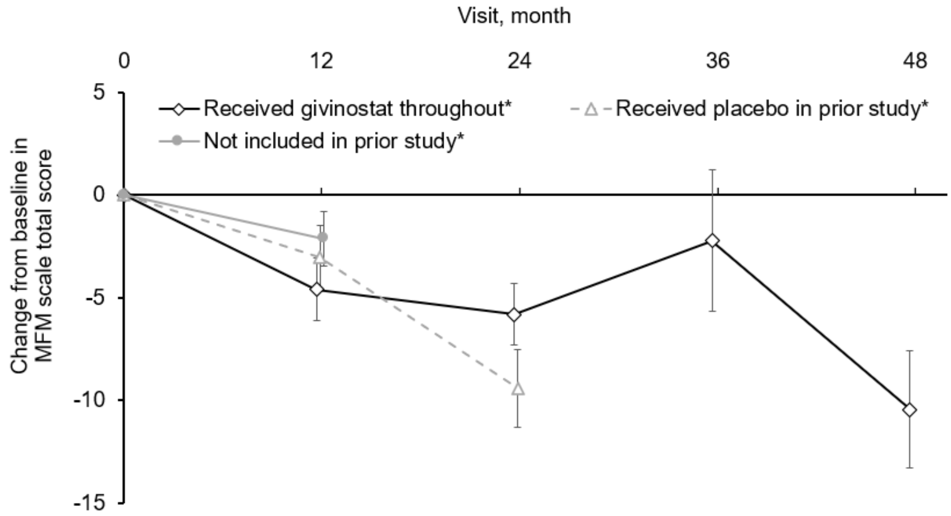

N=					
Givinostat throughout	110	73	43	16	16
Placebo in prior study	54	37	18	†	†
Not included	30	27	†	†	†

*All patients were also receiving systemic corticosteroids for the full duration of the follow-up period. †No patients have reached this timepoint. Data are mean and standard error, with N indicating the number available for assessment. Mean (SD) values at baseline were 75.723 (10.6182), 75.199 (8.4874), and 81.067 (7.9868) in the givinostat throughout, prior placebo, and not included groups, respectively.

Supplementary Figure 20. Change from baseline in Motor Function Measure (MFM) scale standing and transfers score (overall population).

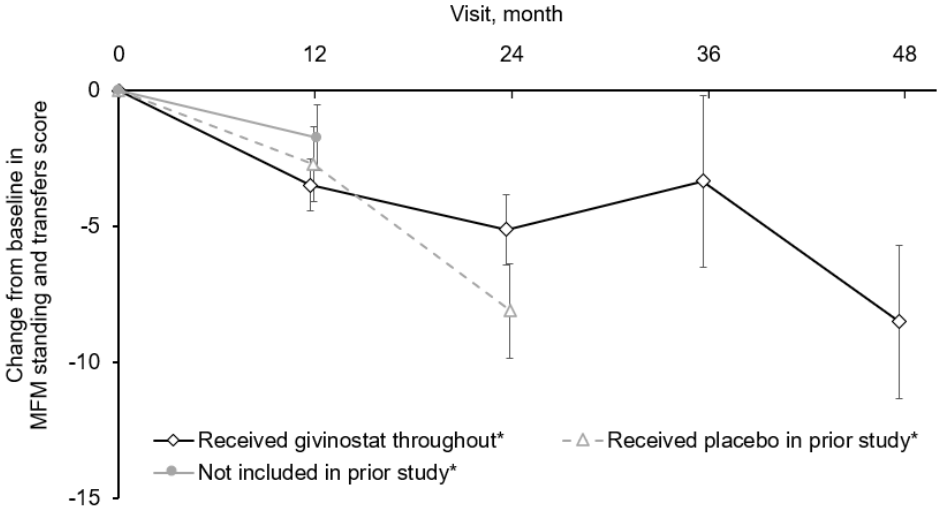

N=					
Givinostat throughout	110	73	43	16	16
Placebo in prior study	54	37	18	†	†
Not included	30	27	†	†	†

*All patients were also receiving systemic corticosteroids for the full duration of the follow-up period. †No patients have reached this timepoint. Data are mean and standard error, with N indicating the number available for assessment. Mean (SD) values at baseline were 21.934 (8.8693), 21.141 (7.3994), and 26.167 (7.4745) in the givinostat throughout, prior placebo, and not included groups, respectively.

Supplementary Figure 21. Change from baseline in Motor Function Measure (MFM) scale axial and proximal motor function score (overall population).

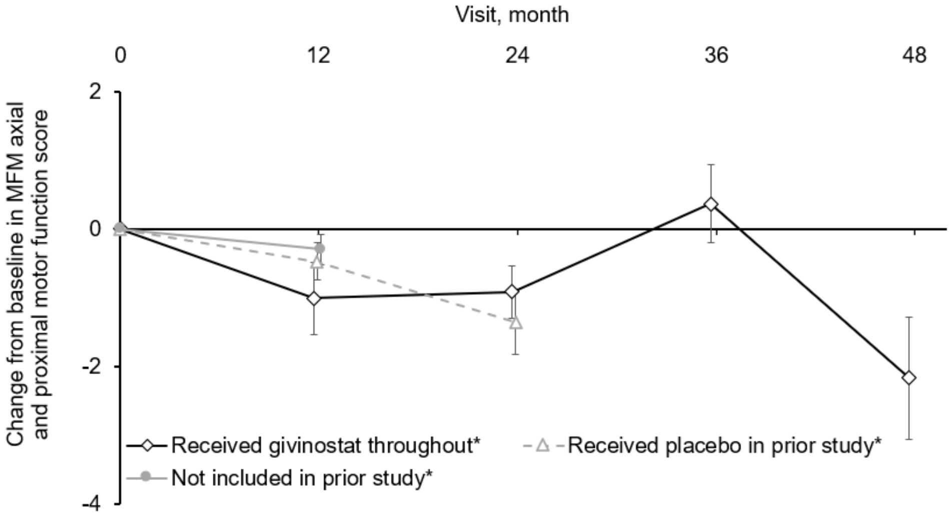

N=					
Givinostat throughout	110	73	43	16	16
Placebo in prior study	54	37	18	†	†
Not included	30	27	†	†	†

*All patients were also receiving systemic corticosteroids for the full duration of the follow-up period. †No patients have reached this timepoint. Data are mean and standard error, with N indicating the number available for assessment. Mean (SD) values at baseline were 34.716 (1.9318), 34.804 (1.6371) and 35.633 (0.5561) in the givinostat throughout, prior placebo, and not included groups, respectively.

Supplementary Figure 22. Change from baseline in Motor Function Measure (MFM) scale distal motor function score (overall population).

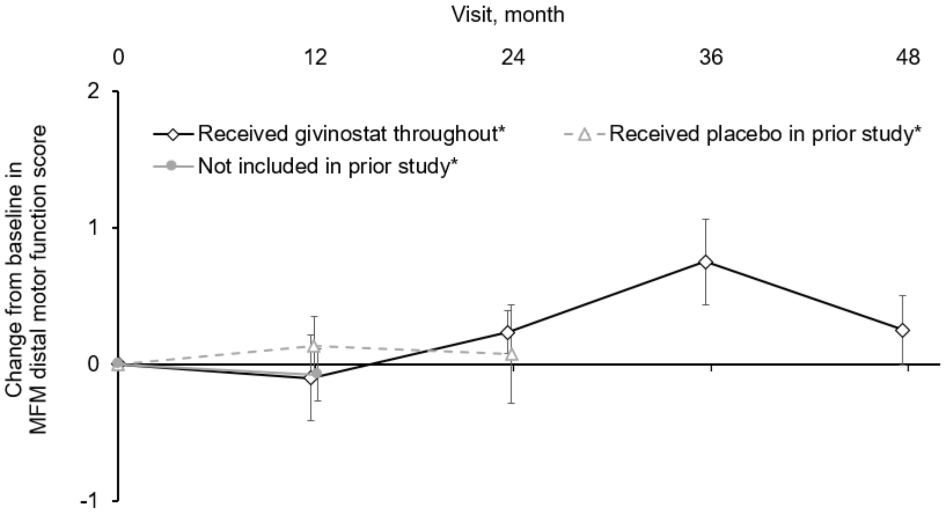

N=					
Givinostat throughout	110	73	43	16	16
Placebo in prior study	54	37	18	†	†
Not included	30	27	†	†	†

*All patients were also receiving systemic corticosteroids for the full duration of the follow-up period. †No patients have reached this timepoint. Data are mean and standard error, with N indicating the number available for assessment. Mean (SD) values at baseline were 19.073 (2.2215), 19.255 (1.2863), and 19.267 (1.2847) in the givinostat throughout, prior placebo, and not included groups, respectively.

Supplementary Figure 23. Change from baseline in forced vital capacity (FVC) percent predicted (overall population).

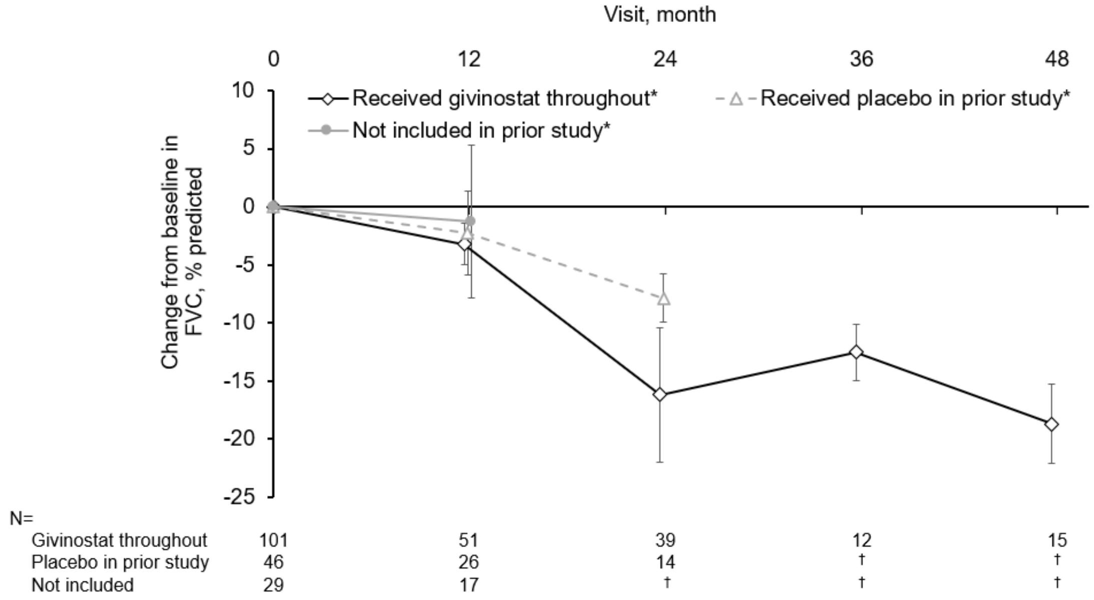

*All patients were also receiving systemic corticosteroids for the full duration of the follow-up period. †No patients have reached this timepoint. Data are mean and standard error, with N indicating the number available for assessment. Mean (SD) values at baseline were 95.76 (28.309), 96.15 (20.859), and 94.71 (29.842) percent in the givinostat throughout, prior placebo, and not included groups, respectively.

Supplementary Figure 24. Change from baseline in peak expiratory flow (PEF) percent predicted (overall population).

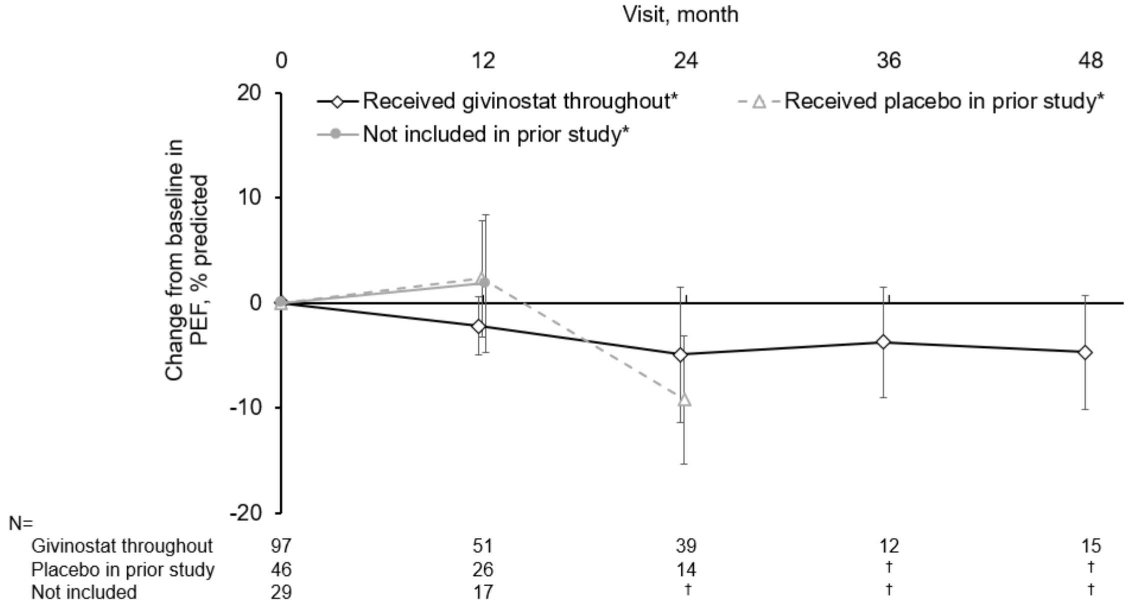

*All patients were also receiving systemic corticosteroids for the full duration of the follow-up period. †No patients have reached this timepoint. Data are mean and standard error, with N indicating the number available for assessment. Mean (SD) values at baseline were 86.90 (31.977), 75.89 (25.694), and 75.93 (28.023) percent in the givinostat throughout, prior placebo, and not included groups, respectively.

Supplementary Figure 25. Change from baseline in Pediatric Quality of Life Inventory (PedsQL) subject total score (overall population).

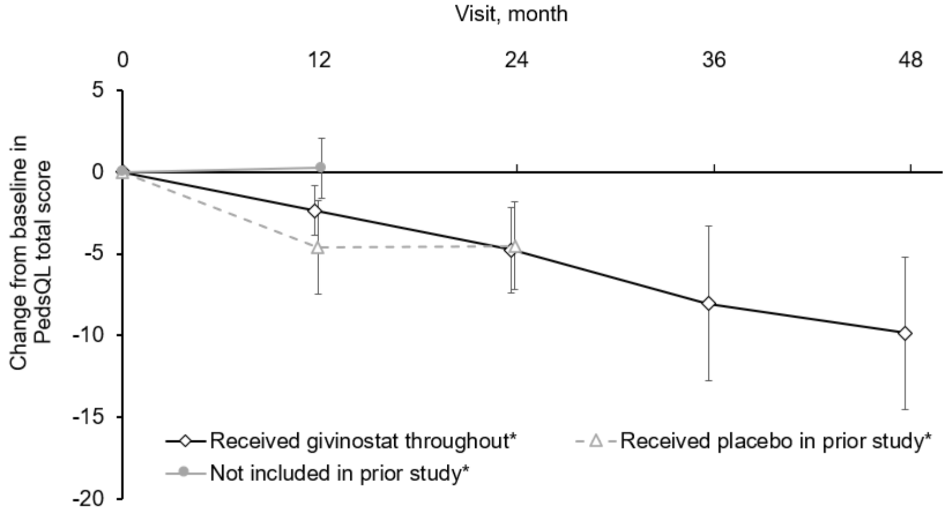

N=	0	12	24	36	48
Givinostat throughout	108	71	43	16	16
Placebo in prior study	50	33	18	†	†
Not included	29	26	†	†	†

*All patients were also receiving systemic corticosteroids for the full duration of the follow-up period. †No patients have reached this timepoint. Data are mean and standard error, with N indicating the number available for assessment. Mean (SD) values at baseline were 65.677 (13.4333), 64.817 (13.6988), and 65.348 (15.9229) in the givinostat throughout, prior placebo, and not included groups, respectively.

Non-ambulant patients

Supplementary Figure 26. Change from baseline in Egen Klassifikation (non-ambulant patients).

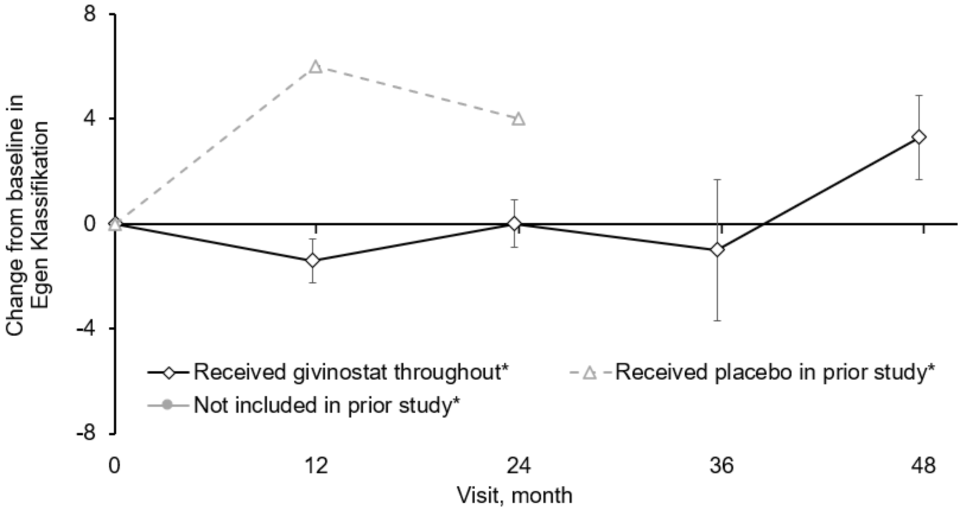

N=	0	12	24	36	48
Givinostat throughout	8	7	5	4	4
Placebo in prior study	3	2	1	†	†
Not included	0	0	0	0	0

*All patients were also receiving systemic corticosteroids for the full duration of the follow-up period. †No patients have reached this timepoint. Data are mean and standard error, with N indicating the number available for assessment. Mean (SD) values at baseline were 7.0 (4.54) and 4.3 (0.58) in the givinostat throughout and placebo groups, respectively.

Supplementary Figure 27. Change from baseline in Barthel Index (non-ambulant patients).

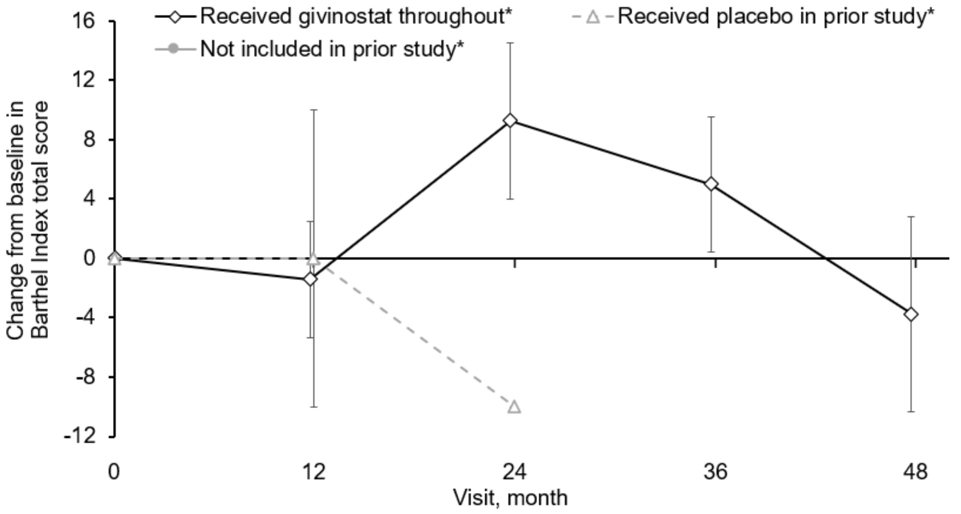

N=	0	12	24	36	48
Givinostat throughout	8	7	5	4	4
Placebo in prior study	3	2	1	†	†
Not included	0	0	0	0	0

*All patients were also receiving systemic corticosteroids for the full duration of the follow-up period. †No patients have reached this timepoint. Data are mean and standard error, with N indicating the number available for assessment. Mean (SD) values at baseline were 46.875 (12.8000) and 66.667 (14.4338) in the givinostat throughout and placebo groups, respectively.

Supplementary Figure 28. Change from baseline in left elbow flexion (non-ambulant patients).

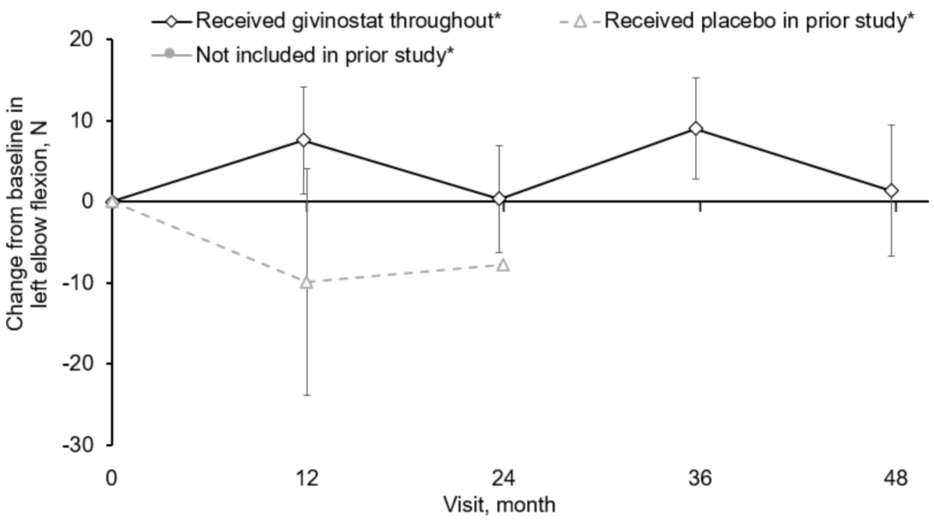

N=	0	12	24	36	48
Givinostat throughout	8	7	5	4	4
Placebo in prior study	3	2	1	†	†
Not included	0	0	0	0	0

*All patients were also receiving systemic corticosteroids for the full duration of the follow-up period. †No patients have reached this timepoint. Data are mean and standard error, with N indicating the number available for assessment. Mean (SD) values at baseline were 24.45 (15.594) and 37.43 (12.158) N in the givinostat throughout and placebo groups, respectively.

Supplementary Figure 29. Change from baseline in right elbow flexion (non-ambulant patients).

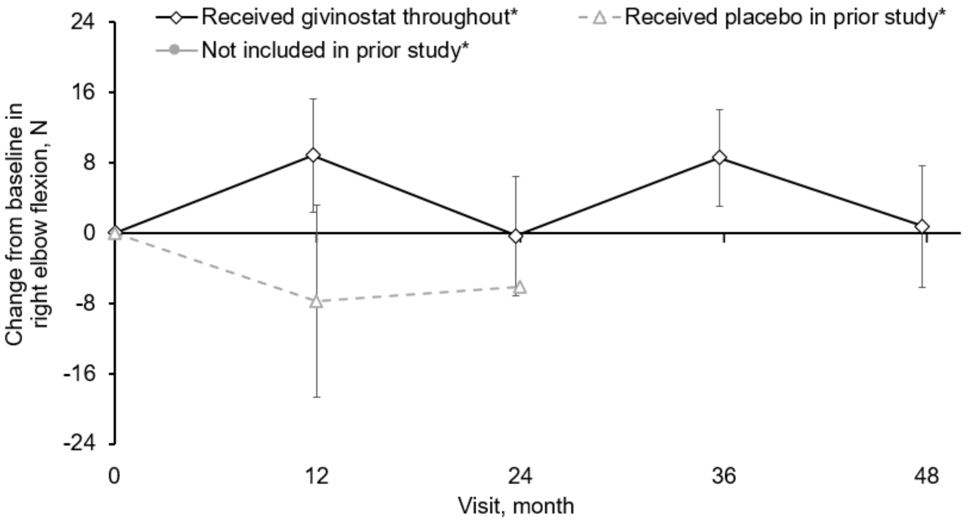

N=	0	12	24	36	48
Givinostat throughout	8	7	5	4	4
Placebo in prior study	3	2	1	†	†
Not included	0	0	0	0	0

*All patients were also receiving systemic corticosteroids for the full duration of the follow-up period. †No patients have reached this timepoint. Data are mean and standard error, with N indicating the number available for assessment. Mean (SD) values at baseline were 24.00 (14.762) and 34.80 (11.776) N in the givinostat throughout and placebo groups, respectively.

Comparison of disease progression milestones with natural history datasets

Supplementary Table 4. Demographic and baseline characteristics of patients included in the full populations from the givinostat study and the natural history datasets.

	Givinostat* (N=148)	Natural history cohort (N=197)
Age, years, mean (SD)	9.9 (2.08)	7.9 (1.66)
Race, n (%)		
Asian	6 (4.1%)	13 (6.6%)
Black	4 (2.7%)	1 (0.5%)
White	128 (86.5%)	111 (56.3%)
Other/unknown	10 (6.8%)	10 (5.1%)
Missing†	0	62 (31.5%)
Body-mass index, kg/m^2, mean (SD)	19.8 (4.08)	18.3 (3.52)
Time since diagnosis†, years, mean (SD)	5.7 (2.69)	4.4 (1.88) (N=62)
Use of corticosteroids, n (%)		
Deflazacort	118 (79.7%)	94 (47.7%)
Other	30 (20.3%)	101 (51.3%)
Time to rise from floor, sec, mean (SD)	6.6 (6.71)	5.0 (1.75)
4 stair climb, sec, mean (SD)	3.6 (1.25)	3.6 (1.21)
Time to walk/run 10 m, sec, mean (SD)	8.3 (33.60)	5.4 (1.58)
6 min walk distance,† m, mean (SD)	396 (71.0)	389 (56.3) (N=58)

*All patients were also receiving systemic corticosteroids for the full duration of the follow-up period. †Information on race is not available from ImagingDMD, with information on time since diagnosis and 6 min walk distance not available from CINRG.

Supplementary Table 5. Comparison of the occurrence of major milestones in the givinostat and natural history datasets – analyses including the full populations.

	**Givinostat*<br>(N=148)**	**Natural history cohort<br>(N=197)**
Persistent loss of rise from floor, n (%)	49 (33.1%)	76 (38.6%)
Age, years, median (95% CI)	14.9<br>(13.4, 15.4)	12.7<br>(12.2, 14.2)
Hazard ratio givinostat vs. control (95% CI)	0.68 (0.47, 0.97); p=0.034	
Persistent loss of 4 stair climb, n (%)	23 (15.5%)	62 (31.5%)
Age, years, median (95% CI)	17.2<br>(15.7, NE)	13.9<br>(13.5, 14.7)
Hazard ratio givinostat vs. control (95% CI)	0.42 (0.26, 0.67); p<0.001	
Persistent loss of ambulation, n (%)	16 (10.8%)	44 (22.3%)
Age, years, median (95% CI)	18.1<br>(18.1, NE)	15.4<br>(14.7, 18.3)
Hazard ratio givinostat vs. control (95% CI)	0.48 (0.27, 0.86); p=0.014	

*All patients were also receiving systemic corticosteroids for the full duration of the follow-up period. n values are the number of patients reaching the disease milestone. NE, not estimable. The p values in this table are nominal.

Supplementary Table 6. Demographic and baseline characteristics, matching patients from the givinostat study population and the natural history datasets – subgroup of patients receiving deflazacort.

	**Givinostat* (N=114)**	**Natural history cohort* (N=92)**
Age, years, mean (SD)	10.1 (2.09)	8.0 (1.70)
Race, n (%)		
Asian	5 (4.4%)	6 (6.5%)
Black	2 (1.8%)	1 (1.1%)
White	98 (86.0%)	43 (46.7%)
Other/unknown	9 (7.9%)	5 (5.4%)
Missing†	0	37 (40.2%)
Body-mass index, kg/m^2, mean (SD)	19.6 (3.88)	18.1 (3.77)
Time since diagnosis†, years, mean (SD)	6.1 (2.57)	4.6 (1.67) (N=37)
Use of corticosteroids, n (%)		
Deflazacort	114 (100%)	92 (100%)
Other	0	0
Time to rise from floor, sec, mean (SD)	5.5 (1.92)	4.7 (1.42)
4 stair climb, sec, mean (SD)	3.3 (1.12)	3.6 (1.11)
Time to walk/run 10 m, sec, mean (SD)	5.2 (1.10)	5.4 (1.66)
6 min walk distance,† m, mean (SD)	409 (65.3)	391 (60.6) (N=35)

*All patients were also receiving systemic corticosteroids for the full duration of the follow-up period. †Information on race is not available from ImagingDMD, with information on time since diagnosis and 6 min walk distance not available from CINRG.

Supplementary Table 7. Comparison of the occurrence of major milestones, matching patients from the givinostat study population and the natural history datasets – subgroup of patients receiving deflazacort.

	**Givinostat*<br>(N=114)**	**Natural history cohort*<br>(N=92)**
Persistent loss of rise from floor, n (%)	32 (28.1%)	30 (32.6%)
Age, years, median (95% CI)	15.2<br>(13.7, 16.4)	14.7<br>(13.2, 15.9)
Hazard ratio givinostat vs. control (95% CI)	0.88 (0.54, 1.44); p=0.606	
Persistent loss of 4 stair climb, n (%)	11 (9.6%)	28 (30.4%)
Age, years, median (95% CI)	18.1<br>(17.2, NE)	14.7<br>(13.8, 15.9)
Hazard ratio givinostat vs. control (95% CI)	0.32 (0.16, 0.64); p=0.001	
Persistent loss of ambulation, n (%)	6 (5.3%)	18 (19.6%)
Age, years, median (95% CI)	18.1<br>(18.1, NE)	15.9<br>(15.0, NE)
Hazard ratio givinostat vs. control (95% CI)	0.36 (0.15, 0.83); p=0.017	

*All patients were also receiving systemic corticosteroids for the full duration of the follow-up period. n values are the number of patients reaching the disease milestone. NE, not estimable. The p values in this table are nominal.
